# Impact of neighborhood socioeconomic status, income segregation, and greenness on blood biomarkers of inflammation

**DOI:** 10.1016/j.envint.2022.107164

**Published:** 2022-03-05

**Authors:** Hari S. Iyer, Jaime E. Hart, Peter James, Elise G. Elliott, Nicole V. DeVille, Michelle D. Holmes, Immaculata De Vivo, Lorelei A. Mucci, Francine Laden, Timothy R. Rebbeck

**Affiliations:** a Division of Population Sciences, Dana-Farber Cancer Institute, Boston, USA; b Department of Epidemiology, Harvard T. H. Chan School of Public Health, Boston, USA; c Channing Division of Network Medicine, Brigham and Women’s Hospital and Harvard Medical School, Boston, USA; d Department of Environmental Health, Harvard T. H. Chan School of Public Health, Boston, USA; e Department of Population Medicine, Harvard Medical School and Harvard Pilgrim Healthcare, Boston, USA

**Keywords:** Biomarkers, Health behavior, Residence characteristics, Socioeconomic factors, Inflammation, Disparities

## Abstract

**Background:**

Neighborhood deprivation is linked with inflammation, which may explain poorer health across populations. Behavioral risk factors are assumed to largely mediate these relationships, but few studies have examined this. We examined three neighborhood contextual factors that could exert direct effects on inflammation: (1) neighborhood socioeconomic status, (2) an index of concentration at extremes (that measures segregation), and (3) surrounding vegetation (greenness).

**Methods:**

Using blood samples and addresses collected from prospective cohorts of 7,930 male (1990–1994) and 16,183 female (1986–1990) health professionals with at least one inflammatory marker, we prospectively linked neighborhood contextual factors to inflammatory biomarkers (adiponectin, C-reactive protein, interleukin-6, soluble tumor necrosis factor receptor-2). Log-transformed, z-scaled component measures were used to calculate an inflammation score. Neighborhood socioeconomic status and index of concentration of extremes were obtained from the 1990 decennial census and linked to participant addresses. Surrounding greenness was assessed from satellite data and focal statistics were applied to generate exposures within 270 m and 1230 m of the participants’ address. We fit multiple linear regression models adjusting for demographic, clinical, and behavioral risk factors.

**Results:**

Higher neighborhood socioeconomic status was associated with lower inflammation score in women (β for interquartile range increase = –27.7%, 95% CI: –34.9%, –19.8%) and men (β = –21.2%, 95% CI: –31.0%, –10.1%). Similarly, participants in neighborhoods with higher concentrations of high-income households were associated with lower inflammation score in women (β = –27.8%, 95% CI: –35.8%, –18.7%) and men (β = −16.4%, 95% CI: –29.7%, –0.56%). Surrounding greenness within 270 m of each participant’s address was associated with lower inflammation score in women (β = −18.9%, 95% CI: –28.9%, –7.4%) but not men. Results were robust to sensitivity analyses to assess unmeasured confounding and selection bias.

**Discussion:**

Our findings support the hypothesis that adverse neighborhood environments may contribute to inflammation through pathways independent of behavioral risk factors, including psychosocial stress and toxic environments.

## 1. Introduction

Humans have long recognized that the places where people live influence their health, with records dating back to the fourth century BCE (Duncan et al., 2018). Neighborhood contextual factors, encompassing socioeconomic, demographic, and environmental features of areas surrounding peoples’ residences, have been linked with cardiovascular disease, diabetes, and many cancers (Roux et al., 2001; Malambo et al., 2016; Gomez et al., 2015; Bilal et al., 2018). However, translating this evidence into interventions has proven difficult, in part because mechanistic pathways linking neighborhood environmental exposure to pathological processes remain poorly understood (Duncan et al., 2018). Clarifying the relevant multilevel pathways that underlie associations between neighborhood environments and health is necessary for informing policies to improve neighborhoods and health of their residents (Roux and Mair, 2010).

**Fig. 1.**
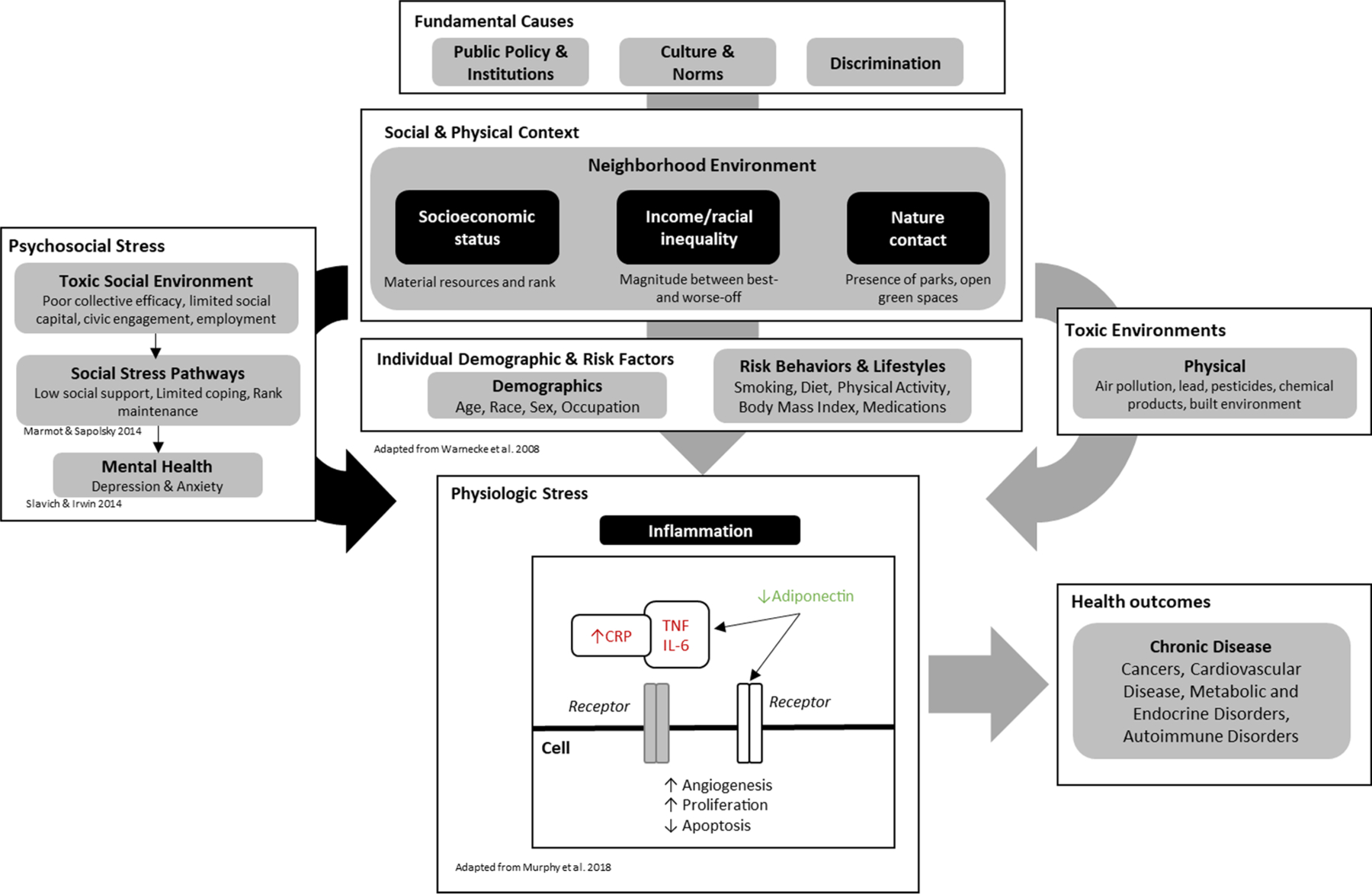
Conceptual framework linking neighborhood environments to health outcomes via stress-related, demographic, environmental, and physiological pathways.

Fig. 1 presents a conceptual model, based on Warnecke et al.’s model for organizing multilevel factors that contribute to health disparities (Warnecke et al., 2008). This model describes mechanistic pathways linking neighborhood environments to inflammation, which contributes to the development of numerous chronic diseases including cardiovascular disease and cancers (Lopez-Candales et al., 2017 Apr; Greten and Grivennikov, 2019). For example, neighborhood socioeconomic status (nSES) captures the educational, occupational, and wealth composition of a given area, as well as the material resources available to residents (Krieger et al., 1997). Income inequality, which captures the absolute and relative differences between those at the highest and lowest ends of the income distribution in a given population, has also been linked with poorer mental and physical health (Kawachi et al., 2014). The Index of Concentration at Extremes (ICE), which captures the homogeneity of a given neighborhood with respect to, for example, income and race, has been linked with higher rates of chronic disease, cancer, and hypertension, and greater exposure to air pollution (Krieger et al., 2018; Krieger et al., 2018; Krieger et al., 2015). Growing evidence suggests that living in environments containing higher levels of vegetation (i.e., “greenness”) is linked with better mental health and stress reduction, and lower incidence of numerous health outcomes including cardiovascular disease, diabetes, and all-cause mortality (James et al., 2015; Jimenez et al., 2021; Fong et al., 2018; Rojas-Rueda et al., 2019).

There are three important pathways through which neighborhood environments may influence biological processes that lead to ill health (Kubzansky et al., 2014). One pathway is through psychosocial stress, caused by toxic social environments. Examples of toxic social environments include poor social cohesion, and limited social capital, civic engagement, and employment, which are more prevalent in socioeconomically deprived neighborhoods (Steptoe and Feldman, 2001) and possibly mediated through limited green space (Jennings and Bamkole, 2019). These exposures can contribute to adverse physical and mental health through low social support, limited coping, and frustration with lack of opportunities for social mobility (Marmot and Sapolsky, 2014). Second, individual-level demographic and behavioral risk factors may also serve as mediators of neighborhood environments and health. Examples include smoking, diet, physical activity, body mass index, and medications (Pollitt et al., 2007). Finally, toxic physical environmental factors, including air pollution, lead, and chemical pollutants, which often are more prevalent in low income neighborhoods composed primarily of people of color (Pastor et al., 2005; Rachel et al., 2006), also can lead to poorer health (Brauer et al., 2021).

Together, psychosocial stress, behavioral risk factors, and toxic environments may all contribute to physiologic stress (Kubzansky et al., 2014). There is a growing focus on stress-related pathways leading to inflammation. Inflammation exerts pathological effects across organ systems that lead to many chronic diseases, including cardiovascular disease, diabetes complications, and many cancers. Toxic social environmental stressors exert physiologic changes via the hypothalamus–pituitaryadrenal (HPA) axis, stimulating sympathetic and adrenal hormone release of inflammatory cytokines and reducing adipokines (Irwin and Cole, 2011; Murphy et al., 2018). Sustained signaling through these pathways results in chronic inflammation, which may lead to higher risk of cardiovascular disease, cancer, and other conditions (Irwin and Cole, 2011). Higher levels of inflammatory markers such as C-reactive protein (CRP), interleukin-6 (IL-6), soluble tumor necrosis factor (sTNFR-2), and lower levels of adipokines may cause cells to adopt several “hallmarks of cancer” (Hanahan and Weinberg, 2011), including increased angiogenesis, cellular proliferation, and escape from apoptosis (Irwin and Cole, 2011; Murphy et al., 2018). These pathways may interact with underlying individual-level comorbidities to promote carcinogenesis (Murphy et al., 2018; Giovannucci, 2018). Higher levels of CRP and IL-6 in residents of low nSES neighborhoods support the hypothesis that neighborhood deprivation may influence disease through inflammatory pathways (Milaniak and Jaffee, 2019; Muscatell et al., 2020; Liu et al., 2017).

We leveraged data from case-control studies nested within two cohorts of male and female health professionals in the United States to examine the strength of associations between nSES, neighborhood income ICE, and neighborhood greenness with blood biomarkers of inflammation. These neighborhood factors were chosen because their health effects may be mediated by inflammatory pathways. We hypothesized that higher nSES, income ICE, and neighborhood greenness exposure would be associated with lower (more favorable) inflammation profile.

## 2. Materials and Methods

### 2.1. Study design and population

We conducted this study using data collected as part of two ongoing nation-wide prospective cohort studies. The Nurses’ Health Study (NHS) is made up of 121,700 female registered nurses. The Health Professionals Follow-up Study (HPFS) is made up of 51,529 male health professionals. In NHS, 32,826 women provided blood samples between 1989 and 1990, while in HPFS, 18,000 men provided samples between 1993 and 1995. Samples were used for nested case-control studies to prospectively assess impact of plasma biomarkers on cancer, cardiovascular, and neurodegenerative disease risk. Risk set sampling was used to identify controls. Participants with prior history of cancer, cardiovascular disease, and diabetes were not eligible for inclusion in these blood biomarker studies. Residential addresses were obtained from questionnaires in NHS in 1986, and every two years thereafter. Similarly, in HPFS, addresses were collected beginning in 1988 and biennially thereafter, and were a mix of home or place of work. If men reported an address that was both home and work, we classified that address as home. In order to standardize the exposure window across neighborhood contextual factors, we estimated exposures for all addresses for the four years prior to blood draw (NHS: 1986–1990; HPFS: 1990–1994).

In NHS, we identified 17,872 women with inflammatory biomarkers by pooling data across multiple nested case-control studies. Participants with erroneous records (n 16) and outlier values defined using the generalized extreme studentized deviate procedure (Rosner, 1983) for adiponectin (n = 4), CRP (n = 100), IL-6 (n = 86), and sTNFR-2 (n = 49), along with those with missing geospatial data (n = 14) and a prior history of cancer, cardiovascular disease, or diabetes prior to blood draw (n = 1,420) were excluded. In HPFS, we identified 9,167 men with inflammatory biomarkers and then excluded participants with outlier values for adiponectin (n = 43), CRP (n = 100), IL-6 (n = 80), and sTNFR-2 (n = 13), as well as those missing geospatial data (n 5) or with a prior history of chronic disease (n = 996). We obtained a final analytic sample of 16,183 participants from NHS and 7,930 participants from HPFS with at least one inflammatory biomarker.

### 2.2. Neighborhood socioeconomic status

Neighborhood socioeconomic status was assessed using a composite score derived separately in NHS and HPFS. Data used to generate the nSES score were obtained from the United States 1990 Decennial Census and linked to participants’ addresses during the four years prior to blood draw. The nSES score includes census tract level variables for educational attainment (% over 25 with college or higher education), income (median family income), wealth (median family home value, % families receiving interest dividends or rent income, % occupied housing units), employment status (% population 16 + years old unemployed), and racial composition (% White, % Black, % foreign-born) (Krieger et al., 1997). We calculated a study-specific summary index of nSES by z-scaling each component measure and then summing across the nine indicators. We checked the distribution of census tract-level poverty and income measures in the NHS and HPFS against census tract measures across the total population of the US and found remarkable similarities (Supplementary Fig. 1).

### 2.3. Index of Concentration at the Extremes

The Index of Concentration at the Extremes (ICE) is a measure of “social spatial polarization” that facilitates comparisons between neighborhoods exhibiting a high degree of racial and socioeconomic segregation (Krieger et al., 2016). The measure is computed by taking the difference in total number of people occupying an advantaged vs disadvantaged position in society with respect to race (White vs Black) and income (highest vs lowest quintile of income) and dividing by the total number of people in that geographic area.Values range from –1 to 1, with values of –1 indicating a uniformly segregated disadvantaged area (for example, all low-income), and 1 indicating a uniformly segregated advantaged area (for example, all high-income). We calculated 1990 census tract-level measures of income and joint race-income ICE for the contiguous United States and linked to participant addresses in the four years prior to blood draw.

Assessments of quintiles of income were based on the distribution of 1990 census household income. We summed the number of White, Black, American Indian, Asian, Native Hawaiian and other Pacific Islander, Other race households, and Hispanic/Latino households reporting ≥$50,000 or more income in the prior year (highest income quintile) and those reporting ≤$9,999 (lowest income quintile). The difference between these two quantities (numerator) was divided by the total population in that census tract (denominator) to calculate the Income ICE. For the joint race-income ICE measure, the numerator was the difference in the number of White households reporting ≥$50,000 or more income in the prior year (White households in highest income quintile) and Black households reporting ≤$9,999 (Black households in the lowest income quintile).

### 2.4. Neighborhood greenness

Exposure to neighborhood greenness was assessed using the Normalized Difference Vegetation Index (NDVI), a satellite-derived measure that captures photosynthetic activity of green, leafy vegetation (Kriegler et al., 1969). Values range from –1 to 1, with values between 0 and 1 corresponding to increasing levels of green leafy vegetation. Values below 0 indicate clouds, snow, or water. Data were obtained from the Landsat 5 and 7 satellites, which captured NDVI at 30 m resolution from 1984 through 2014 using Google Earth Engine (Gorelick et al., 2017). We applied Google Earth Engine’s cloud cover algorithm to retain the least cloudy image within each season (January-March, April-June, July-September, October-December) from 1986 through 1994, capturing the four-year window at all addresses prior to blood draw across both cohorts.

Greenness may influence inflammation pathways through physical and psychosocial pathways that operate at different spatial scales38. We therefore chose to approximate greenness exposures at the level of the immediate environment around the participant’s address, and at a larger neighborhood-level scale. These two spatial scales would capture potential benefits of seeing and spending time in residential areas with high surrounding greenness, vs interacting with public green spaces. We calculated focal statistics using a 270 meter (270m) circular buffer and 1230 meter (1230m) circular buffer using the reduceNeighborhood, ee. Reducer.mean, and ee.Kernel.circle functions in Google Earth Engine.

We hypothesized that greenness exposure accumulated over time prior to blood draw, rather than contemporaneous greenness exposure, would be more strongly associated with lower inflammation. Our primary greenness exposure measure was modeled using a seasonal average of NDVI measures that were available during the four years prior to blood draw for both cohorts (NHS: 1986–1990; HPFS: 1990–1994.As a sensitivity analysis, we also obtained measures of seasonal greenness levels at participant’s addresses during the month of blood draw. Prior to calculating our greenness exposure measures, we set NDVI values that were either missing or < 0 to 0. NDVI measures were then assigned to participant addresses.

### 2.5. Inflammatory biomarkers

To examine associations between neighborhood contextual exposures and inflammatory pathways, we selected a set of biomarkers related to inflammatory response (adiponectin, CRP, IL-6, sTNFR-2). Inflammatory biomarkers were extracted from blood samples provided by study participants. Those who agreed to participate were sent a blood collection kit containing supplies for drawing, storing, and shipping the sample, along with detailed instructions. Blood samples were collected remotely by each participant in treated tubes, placed on ice, stored in styrofoam containers and shipped overnight to the laboratory. Blood samples were then centrifuged, divided into aliquots and stored in liquid-nitrogen freezers. Case and control samples were run together for all assays, and laboratory staff were blinded to case status of samples. The enzyme-linked immunosorbent assay/radioimmunoassay (ELISA/RI from ALPCO Diagnostics (Heidemann et al., 2008) was used to assess adiponectin. The latex-enhanced immunoturbidimetric assay (LEI) with reagents and calibrators from Denka Seiken was used to assess CRP. ELISA was used to assess IL-6 and sTNFR-2. Random quality-control samples were run with each batch of case-control samples. Quality-control sample intra-assay CV ranged from 1% to 20% across all batches of inflammatory markers. Summary statistics for measured inflammatory markers by cohort and laboratory batch are available in Supplementary Table 1. Further details regarding the assays are reported elsewhere (Hang et al., 2019).

In order to assess associations between neighborhood environmental factors and overall inflammation, we calculated a summary inflammatory score using the approach of Tabung et al. (Eq. (1)) (Tabung et al., 20162016):

$$zscore\left(\log\left(IL-6\right)\right)+zscore\left(\log\left(CRP\right)\right)+zscore\left(\log\left(TNF\alpha R2\right)\right)-zscore\left(\log\left(Adiponectin\right)\right)$$

Although this score was initially designed to capture diet-induced inflammation, the score does not explicitly incorporate diet-based weighting but rather simply summarizes the overall relationship between CRP, IL-6, sTNFR-2, and adiponectin. This global summary score complements findings from investigations of individual inflammation biomarkers by providing a composite estimate of blood levels of inflammation. We accounted for variability in mean stress biomarker levels arising from laboratory procedures, technicians, and time of collection by applying Rosner’s method for standardizing within-batch mean values for a given biomarker to the global mean for that biomarker, reflecting the value of an “average batch.” (Rosner et al., 2008).

### 2.6. Statistical analysis

We examined correlations between independent and dependent variables using Spearman’s correlation to account for the skewed distribution of biomarker variables. All biomarker measures were log-transformed prior to regression modeling to account for skewness. Comparing correlations between variables in men and women separately revealed different patterns of correlation between NDVI, stress biomarkers, and covariate data and so we chose to present results stratified by sex.

For our primary analysis, we fit multiple linear regression models to estimate the association between neighborhood contextual variables and our stress biomarker endpoints. Beta coefficients were transformed to represent a percentage change in biomarker associated with a change in exposure. Deviations from linearity for dose–response relationships between each exposure and outcome were assessed via restricted cubic splines. As no deviations were observed, associations are presented for an interquartile range (IQR) increase for nSES, NDVI, and ICE measures. All covariates were assessed at blood draw unless otherwise stated. We adjusted for age, fasting status, smoking status (current smokers, former smokers, never smokers), history of hypertension (binary), history of hypercholesterolemia (binary), BMI (<23, 23–<25, 25-<27.5, 27.5-<30, ≥ 30 kg/m^2^), census region (Northeast, Midwest, South, West), population density (<1000 people/mi^2^, 1000 people/mi^2^), post-menopausal hormone use (women only: pre-menopause and missing, post-menopause and never use, current use, past use), case status (binary), and any use of anti-inflammatory medications (binary). In order to account for air pollution exposures, we adjusted for Particulate Matter 2.5 µm in diameter or less (PM_2.5_) using estimates from a spatiotemporal model for the contiguous US (Yanosky et al., 2014). We averaged daily PM_2.5_ estimates at participant addresses over 24 months prior to blood draw (due to the temporal availability of air pollution estimates). We did not mutually adjust for nSES and the ICE measures because they were created from shared input variables, which led to collinearity. We adjusted for continuous nSES in models for NDVI because nSES was assumed to drive exposure to NDVI, rather than the reverse. In the NHS, we considered the following variables as measures of individual SES: mother’s occupation, father’s occupation, husband’s education level, and marital status because these individual-level SES variables were only available in that cohort. Further adjustment for these variables did not lead to changes in our results and so we did not include them in our final models.

To determine robustness of our results to potential bias arising from sample selection and participant neighborhood mobility, we performed multiple sensitivity analyses. We repeated analyses further adjusting for diet quality (Alternative Healthy Eating Index, quintiles) and physical activity (categorical: <3, 3-<9, 9-<18, 18-<27, ≥27 MET-hours/week) to determine whether results were independent of these potential confounding or mediating variables. We restricted analysis to controls only, thereby limiting confounding by emergent disease processes that may have been associated with NDVI and stress biomarkers. We then repeated analyses restricting to participants who did not change addresses during follow-up. Since HPFS participants reported either home or work address, we examined whether associations varied by address type. We also assessed whether associations between NDVI and inflammatory markers varied by census region. All hypothesis tests were two-sided with alpha = 0.05.

## 3. Results

Characteristics of the study population at the time of blood draw by quintiles of nSES (higher indicates increasing neighborhood privilege) are presented in Table 1. In NHS, most women (98.6%) were White, with a median age of 57.4 years. In HPFS, men were also predominantly White (93.8%) with a median age of 62.5 years. Women living in areas with higher nSES were less likely to be never smokers (Q5: 42.6% vs Q1: 48.9%) and had better diet quality as measured by the Alternative Healthy Eating Index (Q5: 55.9 vs Q1: 51.4). Men in higher nSES were more likely to be never smokers (Q5: 46.8% vs Q1: 45.1%) and reported higher diet quality (Q5: 56.6 vs Q1: 51.9). Men and women living in areas with higher nSES also lived in more densely populated areas. Correlations between neighborhood contextual factors, individual diet and lifestyle factors, and inflammatory biomarkers in NHS and HPFS are described in Supplementary Fig. 2. We observed distinct correlation patterns within several groups of variables: (1) NDVI measures, (2) nSES and ICE measures, and (3) the inflammatory biomarkers and BMI.

### 3.1. Associations between neighborhood contextual factors, inflammatory biomarkers and inflammation score

**Fig. 2.**
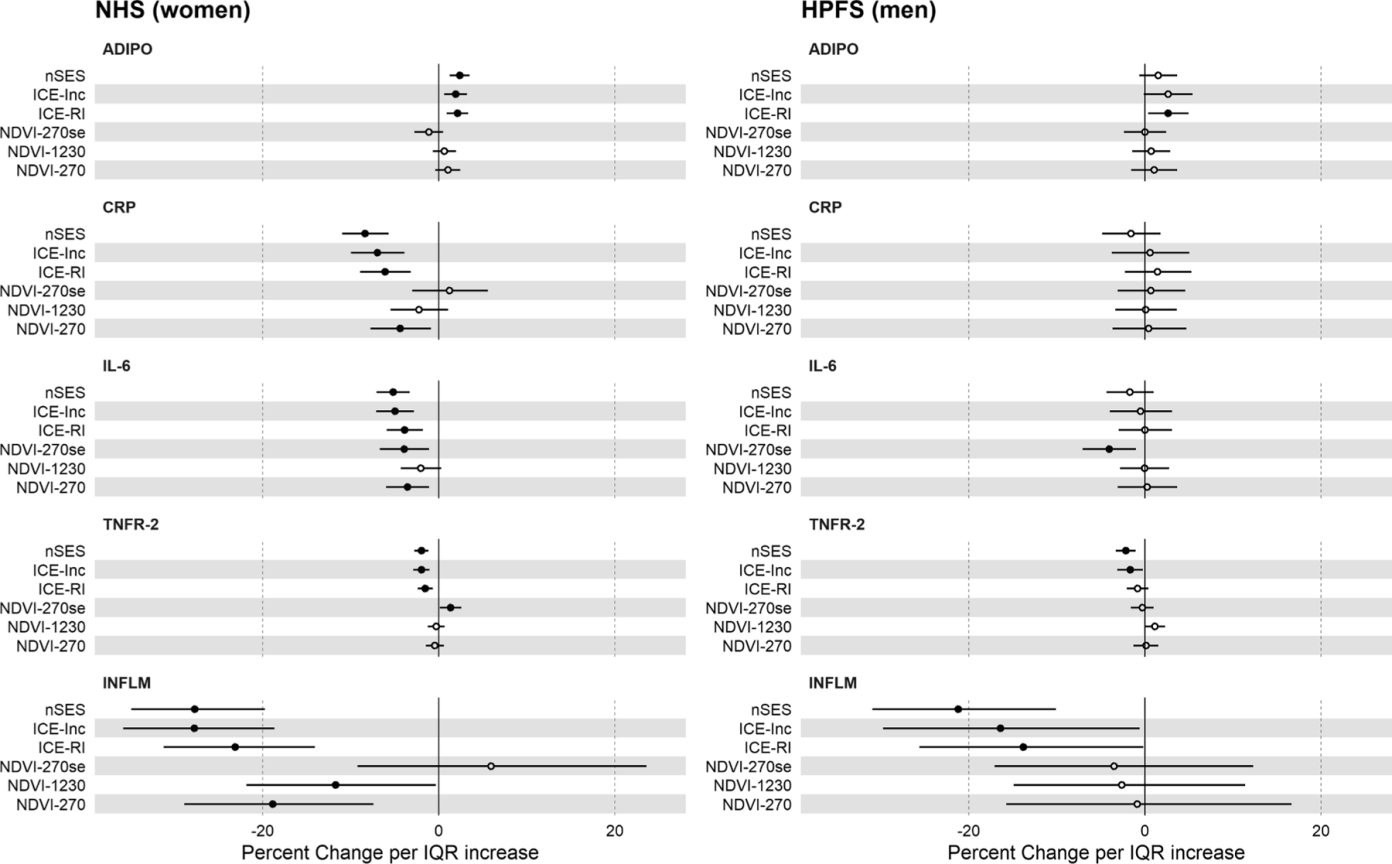
Associations between neighborhood contextual factors and inflammatory blood biomarkers from linear regression models among women and men in the Nurses’ Health Study (n = 16,183 women^a^) and Health Professionals Follow-up Study (n = 7,930 men^a^) Abbreviations: ADIPO: Adiponectin; CRP: C-Reactive Protein; IL-6: Interleukin-6; TNFR-2: soluble tumor necrosis factor receptor-2; INFLM: inflammation score; nSES: Neighborhood Socioeconomic Status; ICE-Inc: Index of Concentration at Extremes-Income; ICE-RI: Index of Concentration at Extremes-Race/Income; NDVI: Normalized Difference Vegetation Index (NHS: 1986–1990; HPFS: 1990–1994), NDVI-270se: 270m seasonal Normalized Difference Vegetation Index. NHS: Nurses’ Health Study; HPFS: Health Professionals Follow-up Study. All variables are scaled to one-interquartile range increase except ICE-measures which are scaled to standard deviation. Multiple linear regression models for inflammatory markers adjusted for age, fasting status, smoking, hypertension, hypercholesterolemia, body mass index, census region, population density, case status, air pollution (PM_2.5_), and use of anti-inflammatory medication. For NHS, models further adjusted for postmenopausal hormone use. Models for association between NDVI and inflammatory biomarkers were adjusted for nSES. ^a^Sample sizes from NHS and HPFS with at least one biomarker. Sample sizes corresponding to models for each inflammatory biomarker endpoint and neighborhood contextual variable are provided in Supplementary Table 2.

Results from covariate-adjusted linear regression models for the association between neighborhood contextual factors and inflammatory biomarkers are presented in Fig. 2, with numerical estimates provided in Supplementary Table 2. In the NHS, an IQR increase in nSES was inversely associated with CRP (β = −8.38%, 95% CI: –10.99%, –5.69%), IL-6 (β = −5.19%, 95% CI: –7.07%, –3.28%), sTNFR-2 (β = −1.95%, 95% CI: –2.74%, –1.14%), and the overall inflammation score (β = −27.73%, 95% CI: –34.91%, –19.76%). In the HPFS, an IQR increase in nSES was inversely associated sTNFR-2 (β = −2.18%, 95% CI: –3.30%, –1.04%) and the overall inflammation score (β = −21.21%, 95% CI: –30.95%, –10.09%). Similar dose–response relationships between neighborhood contextual factors and inflammatory biomarkers were observed using quintiles and *P*_trend_. For example, comparing those in quintile 5 to 1 of nSES, there was a 54.57% (95% CI: –65.12%, –40.81%) lower inflammation score in NHS and 39.46% (95% CI: –56.76%, –15.24%) lower inflammation score in HPFS (Supplementary Table 2).

ICE measures of neighborhood income segregation and race-income segregation were inversely associated with inflammation (Fig. 2, Supplementary Table 2). In the NHS, an IQR increase in ICE-income was associated with lower CRP (β = −6.98%, 95% CI: −9.99%, −3.88%), IL-6 (β = −4.96%, 95% CI: –7.09%, –2.79%), sTNFR-2 (β = −1.97%, –2.89%, –1.05%), and inflammation score (β = −27.75%, 95% CI: –35.83%, –18.66%). An IQR increase in joint ICE-race/income was associated with lower CRP (β = −6.08%, 95% CI: –8.92%, −3.16%), IL-6 (β = −3.86%, 95% CI: –5.87%, −1.80%), sTNFR-2 (β = −1.54%, 95% CI: –2.39%, –0.67%), and inflammation score (β = −23.13%, 95% CI: –31.22%, –14.07%). In the HPFS, associations between ICE-income and ICE-race/income were generally weaker relative to the NHS. An IQR range increase in ICE-income was associated with lower sTNFR-2 (β = −1.68%, 95% CI: –3.13%, –0.20%) and overall inflammation score (β = −16.41%, 95% CI: –29.72%, –0.56%). An IQR range increase in joint ICE-race/income was associated with higher adiponectin (β = 2.65, 95% CI: 0.38%, 4.98%), and lower inflammation score (−13.81%, 95% CI: –25.61%, –0.14%).

Measures of neighborhood greenness (Fig. 2, Supplementary Table 2) were generally inversely associated with inflammatory biomarkers in the NHS but not HPFS. Associations between four-year average NDVI at 270m resolution were more strongly associated with inflammation across the blood biomarkers compared to 1230m and to NDVI measured at season of blood draw. In NHS, an IQR increase in four-year NDVI at 270m was associated with lower CRP (β = −4.36%, 95% CI: –7.74%, −0.87%), IL-6 (β = −3.56%, 95% CI: –5.97%, –1.08%) and overall inflammation score (β = −18.88%, 95% CI: –28.91%, –7.44%). An IQR increase in seasonal NDVI was associated with lower IL-6 (β = −3.90%, 95% CI: –6.66%, –1.06%) and, contrary to expectation, higher sTNFR-2 (β = 1.36%, 95% CI: 0.15%, 2.58%).

### 3.2. Sensitivity analyses for confounding and selection

**Fig. 3.**
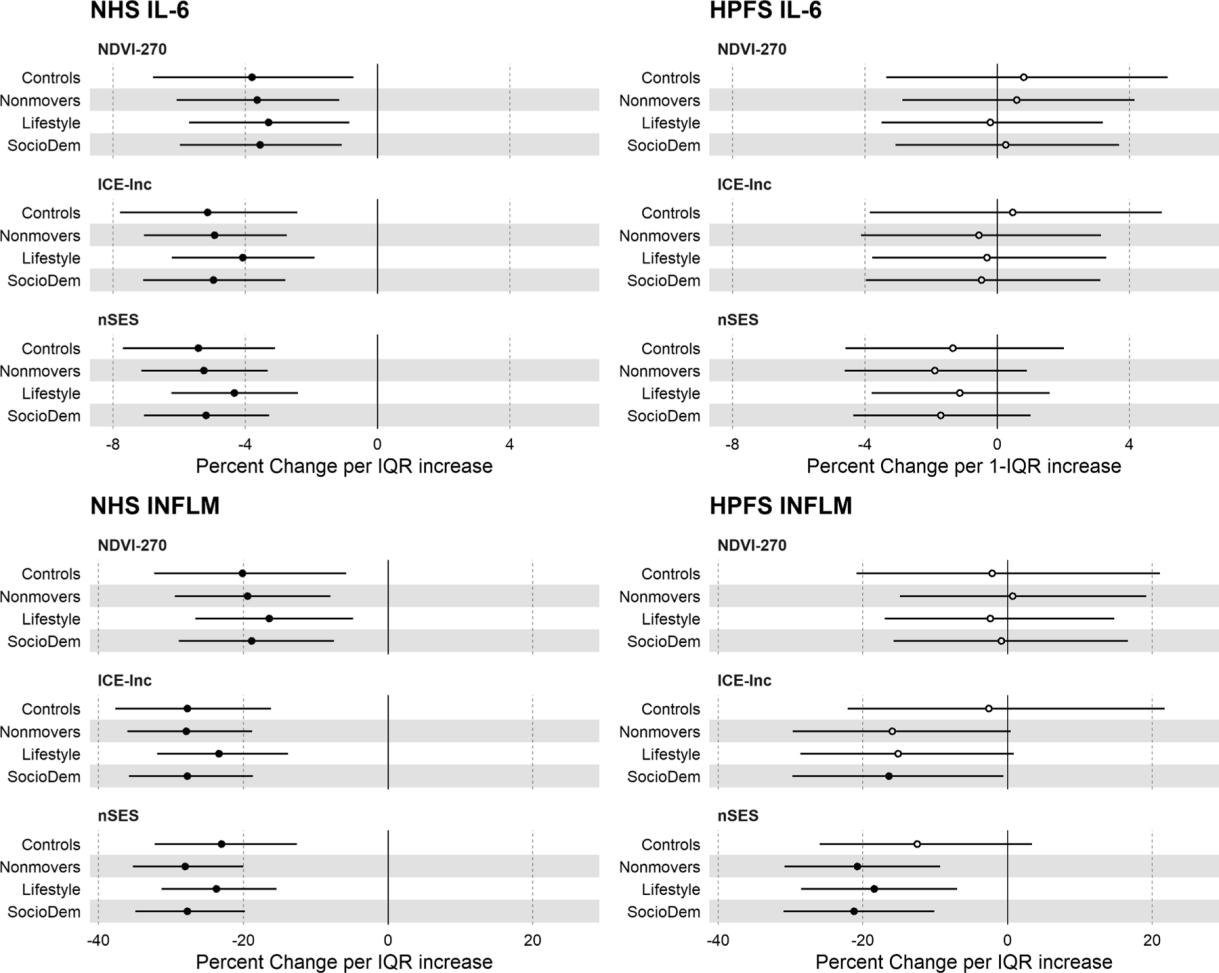
Associations between neighborhood contextual factors, Interleukin-6, and inflammation score from linear regression models following sensitivity analysis among women and men in the Nurses’ Health Study and Health Professionals Follow-up Study Abbreviations: nSES: Neighborhood Socioeconomic Status; ICE-Inc: Index of Concentration at Extremes-Income; NDVI: Normalized Difference Vegetation Index (NHS: 1986–1990, HPFS: 1990–1994), NHS: Nurses’ Health Study; HPFS: Health Professionals Follow-up Study. All variables are scaled to one-interquartile range increase except ICE-measures which are scaled to standard deviation. Multiple linear regression models for inflammatory markers adjusted for age, fasting status, smoking, hypertension, hypercholesterolemia, body mass index, census region, population density, case status, air pollution (PM_2.5_), and use of anti-inflammatory medication. For NHS, models further adjusted for postmenopausal hormone use. Models for association between NDVI and inflammatory biomarkers were adjusted for nSES. Sensitivity analyses included (1) Controls (n = 10,061 for participants with at least one biomarker in NHS, n = 4,481 for HPFS) results from models fit in sampled controls only; (2) Non-movers results from models fit only in those participants who remained at the same address from 1986 (NHS, n = 15,933) or 1988 (HPFS, n = 7,423) through blood draw; (3) Lifestyle models further adjusted for physical activity and diet quality. SocioDem models correspond to main results from Fig. 2. Sample sizes from models for associations between each neighborhood contextual factor and inflammatory biomarker are provided in Supplementary Table 3.

We performed multiple sensitivity analyses to determine robustness of results from regression models by evaluating associations between neighborhood contextual factors, IL-6, and overall inflammation score (Fig. 3, Supplementary Table 3). We further adjusted for individual-level lifestyle factors (physical activity, diet quality) that could be considered confounders or mediators of the association between neighborhood context and inflammation. We also restricted the study population to those sampled as controls (disease-free within the risk set as part of the case-control studies of cardiovascular disease, cancer, and mental health), and separately to those who did not change address over follow-up. Findings from our main analyses were generally robust to these sensitivity analyses (Fig. 3, Supplementary Table 3). In the NHS, the inverse association between an IQR increase in NDVI within 270m of address and overall inflammation score was less precise in controls (β = −20.12%, 95% CI: –32.29%, −5.76%, *P*_trend_ 0.070). In the HPFS, the association between an IQR increase in ICE-income and overall inflammation score was weakened in controls (β = −2.60%, 95% CI: –22.07%, 21.74%) and following adjustment for diet and physical activity (β = −15.14%, 95% CI: 28.61%, 0.87%).

There was no evidence of effect modification of associations between neighborhood contextual factors and inflammatory markers by address type in the HPFS (Supplementary Table 4). We found no evidence of effect modification of associations between NDVI at 270m resolution and inflammatory markers by census region, except for CRP (*P*_het_ = 0.014) and IL-6 (Supplementary Table 5, *P*_het_ = 0.0052). Inverse associations were driven by the Northeast (CRP: −10.14%, 95% CI: −14.82%, −5.21%; IL-6: −4.30%, 95% CI: –7.83%, −0.65%) and South (IL6: −9.27%, 95% CI: –13.84%, –4.46%).

## 4. Discussion

In this study of male and female health professionals, we observed lower levels of inflammatory biomarkers among participants residing in neighborhoods that had more favorable contextual environments. In both cohorts, those with higher nSES exhibited lower overall inflammation. Among participants residing in neighborhoods heavily segregated by income (ICE), those with higher compared to lower concentrations of high-income households had lower inflammatory biomarker profiles, though in the HPFS these associations were not statistically significant. Higher greenness within a 270 m buffer surrounding a participant’s address was associated with lower inflammatory biomarker profiles in the NHS, but not HPFS. Consistency in the direction of associations across multiple measures of neighborhood context and inflammatory biomarkers support the hypothesis that adverse neighborhood context may contribute to poorer health through inflammatory pathways, independent of an individual’s demographics and behavioral risk factors (Fig. 1).

Previous studies have examined associations between neighborhood deprivation and biomarkers of inflammation, though most have focused on objective and perceived nSES only and were conducted in limited geographic areas. Prior cohort studies have examined associations between neighborhood deprivation and inflammation (Pollitt et al., 2007; Pollitt et al., 2008). These studies used prospective designs and controlled for individual-level SES to examine whether perceived stress and neighborhood disorder was associated with greater inflammation among racially diverse populations across several US states. Early childhood SES and adult neighborhood SES were associated with higher CRP, with some evidence of mediation by adult demographic and life-style risk factors (Pollitt et al., 2007). One of the few longitudinal studies of 946 participants in the Multi-ethnic Study of Atherosclerosis reported higher IL-6 over a 3–4 year period among those with lower nSES and lower perceived neighborhood safety (Nazmi et al., 2010). While some studies examined effect modification by race, sex, and other characteristics, patterns were inconsistent, suggesting that mechanistic pathways linking neighborhood environments to health may vary across populations. Recent reviews also find multiple reports supporting a role of inflammation as a mediator of neighborhood deprivation and poorer health, although most studies are cross-sectional (Muscatell et al., 2020; Ribeiro et al., 2018). Our findings from a study using multiple neighborhood measures and a composite index of inflammation provide additional support for the hypothesis that neighborhood context may influence health disparities through inflammation pathways.

We assessed associations between ICE measures and inflammation because place-based health disparities often arise as a result of historical segregationist policies that excluded non-White, low-income populations from certain neighborhoods (Massey, 1993). Using the ICE measure focuses our analysis on how neighborhood inequalities, driven by class and racial hierarchies, may contribute to poorer health in disadvantaged communities. A recent study examining associations between childhood SES and inflammation in Black and White adults found that lower childhood SES was associated with inflammation only among participants who reported lower optimism and purpose in life (Boylan et al., 2020). While detailed longitudinal examinations of effects of social hierarchies on humans are limited, results from animal models demonstrate that disrupting animal housing and social environments can lead to marked changes in biological stress response and gene expression (McClintock et al., 2005; Hermes et al., 2009; Williams et al., 2009; Volden et al., 2013). Evidence from social non-human primates indicates that occupying a lower rank in a social hierarchy leads to high blood pressure and elevated stress hormone levels, slower cardiovascular and endocrine responses to stressors, and a suppressed immune system (Marmot and Sapolsky, 2014). Although results from animal studies cannot be directly extrapolated to humans, common features of primate and human social hierarchies’ effects could shape health responses to repeated stressors.

Our study examined neighborhood socioeconomic and segregation-based measures in a predominantly white, affluent population. Despite limited variability in individual-level SES (race/ethnicity, educational attainment and occupation), the neighborhood socioeconomic measures represented in our cohort were found to reflect that of the US population as a whole (Supplementary Fig. 1). Therefore, our study provides evidence supporting a role of contextual influences of socioeconomic status on blood inflammation pathways that operate independently of individual-level SES. Our findings build on earlier hypotheses and empirical data showing independent individual- and contextual-level impacts of SES on health by testing a proposed biologic pathway that links these upstream contextual factors to health (Pollitt et al., 2007; Pollitt et al., 2008). Confounding by individual-level SES is mitigated through restriction by race/ethnicity, educational attainment, and occupation in our study population. Therefore, these data suggest that social stressors at neighborhood-level, arising from lack of material resources, poor social cohesion, and adverse environmental context may lead to higher blood levels of inflammatory markers regardless of one’s individual ranking in a given social hierarchy (Warnecke et al., 2008; Kawachi and Berkman, 2003; Krieger and Krieger, 2011; McEwen, 1998).

Our study contributes important evidence regarding biological mechanisms through which nature contact influences health. Nature contact may reduce psychosocial stress, which may influence other health outcomes, but specific biological mechanisms remain poorly characterized (Frumkin et al., 2017). Theories of stress reduction and attention restoration arising from nature contact have been proposed as stress-related mechanisms that lead to health benefits (Ulrich et al., 1991; Kaplan and Kaplan, 1989). Experimental studies in East Asian populations have reported physiologic changes associated with nature contact, including lower natural killer cell activity (Li et al., 2008), lower blood pressure, and lower heart rate (Park et al., 2010), but these were conducted in small samples and findings may not be generalizable to other populations. Cross-sectional studies have demonstrated inverse associations between greenness and cardiometabolic biomarkers (Yeager et al., 2018; Yeager et al., 2019). Our study provides additional support for the hypothesis that inflammation may mediate the association between neighborhood greenness and chronic diseases, although we only observed these patterns in women in the NHS. Reasons for this discrepancy could be related to the demographic profiles of these two cohorts (women in the NHS were younger and reflected a broader socioeconomic distribution compared to men in the HPFS) and different locations where neighborhood context was assessed (men reported either home or work addresses). Larger studies with higher spatiotemporal resolution measures of greenness exposure including GPS-based activity spaces could improve understanding of potential differences in associations between greenness and inflammation in men and women (Labib et al., 2020).

Our study has some important limitations. First, our sample relied on data pooled from multiple case-control studies nested within two large cohorts examining a variety of cardiovascular, cancer, and mental health endpoints which could have led to selection bias if case status were differentially associated with neighborhood environmental variables. Reassuringly, our major findings were robust to restriction to controls only, possibly because blood samples were generally taken far before onset of disease. Second, our measures reflect a single address (home or work), which may not reflect the totality of neighborhood contextual exposures encountered by participants over the study period. We averaged neighborhood contextual exposures over time to account for any changes in exposure among participants who moved over follow-up. Restricting to non-movers did not change findings, suggesting that the time periods captured are relevant for inflammation. The summary inflammation score used here may not have fully captured potential inflammation-related pathways specific to the neighborhood contextual factors studied. Use of objective measures of nSES mitigates the potential for differential measurement error, but perceived measures could provide more details regarding the specific chemical and social stressors in the neighborhood environment that drive associations between nSES and inflammation. Unmeasured confounding could have contributed to the associations we observed between our neighborhood contextual factors and inflammation in this observational study. However, our data allowed for detailed control of multiple clinical, demographic, and behavioral risk factors. For example, data on smoking, a major behavioral factor linked with inflammation that was more prevalent at the time the cohorts were enrolled, was readily available. Conducting this study in a fairly affluent, predominantly white population may limit generalizability. However, because the male and female health professionals occupied similar levels of individual SES, residual confounding by SES is unlikely to be a major threat to validity.

### 4.1. Conclusions

In summary, we find support for the hypothesis that neighborhood deprivation is associated with inflammation across nSES, neighborhood income segregation, and neighborhood greenness. These findings are particularly compelling given that they were observed in this narrow spectrum of the population (white health professionals), persisted following adjustment for individual behavioral risk factors, and were consistent across multiple neighborhood contextual factors hypothesized to influence health through inflammation. Our findings can be used to inform individual- and community-level interventions and policies aimed at improving population health.

## Supplementary Material

Supp.materials

## Figures and Tables

**Table 1:** Characteristics of participants with at least one inflammatory biomarker sample available in the Nurses’ Health Study (n = 16,183) and Health Professionals Follow-up Study (n = 7,930) from 1989 to 1995

	Nurses’ Health Study (Women)						Health Professionals Follow-up Study (Men)					
	Quintiles of nSES					Total (n =16183)	Quintiles of nSES					Total (n = 7930)
	Q1 (n = 3236)	Q2 (n = 3237)	Q3 (n = 3238)	Q4 (n = 3238)	Q5 (n = 3234)		Q1 (n = 1586)	Q2 (n = 1586)	Q3 (n = 1587)	Q4 (n = 1585)	Q5 (n = 1586)	
*Demographic characteristics*												
White race, %	99.0	99.1	98.8	98.4	98.1	98.6	94.9	93.2	93.2	94.2	93.2	93.8
Age at blood draw*	58.2 (6.9)	57.5 (7.1)	57.3 (7.0)	58.9 (7.0)	57.0 (6.9)	57.4 (7.0)	63.1 (8.5)	62.5 (8.5)	62.1 (8.6)	62.2 (8.3)	62.6 (8.6)	62.5 (8.5)
*Individual lifestyle factors*												
Body Mass Index	26.0 (5.0)	25.9 (4.8)	25.7 (4.7)	25.4 (4.5)	24.7 (4.2)	25.5 (4.7)	26.0 (3.3)	25.9 (3.2)	25.9 (3.2)	25.7 (3.6)	25.5 (3.0)	25.8 (3.3)
Physical activity (MET-hrs/week)	41.4 (110.0)	38.9 (108.3)	42.1 (113.9)	41.4 (113.5)	36.9 (98.7)	40.4 (110.0)	32.5 (26.7)	31.7 (26.4)	32.6 (26.0)	31.6 (25.7)	30.7 (24.2)	31.8 (26.1)
Smoking pack-years	12.0 (18.5)	11.6 (18.0)	12.4 (18.7)	13.7 (19.3)	12.4 (18.6)	12.4 (18.6)	14.2 (19.4)	13.3 (19.1)	11.9 (17.9)	11.4 (16.8)	11.4 (16.5)	12.5 (18.3)
Smoking status												
Never smoker, %	48.9	49.1	45.4	40.3	42.6	45.3	45.1	47.7	49.5	47.9	46.8	47.0
Past smoker, %	37.6	37.3	41.3	44.9	45.4	41.2	46.6	45.7	45.2	46.8	48.9	47.2
Current smoker, %	13.5	13.6	13.3	14.8	12.0	13.5	8.3	6.7	5.3	5.2	4.4	5.8
Alternative Healthy Eating Index	51.4 (10.1)	52.1 (9.9)	53.1 (10.4)	53.8 (10.2)	55.9 (10.2)	53.3 (10.2)	51.9 (10.3)	52.9 (10.4)	53.9 (10.6)	55.1 (10.2)	56.6 (10.7)	54.1 (10.7)
Menopause												
Premenopause, %	18.5	18.6	19.1	19.0	19.8	19.0						
Postmenopause, %	74.2	74.5	74.5	74.3	74.7	74.5						
Dubious menopause and missing, %	7.3	7.0	6.4	6.7	5.5	6.5						
Hormone therapy												
Never use, %	35.9	36.5	37.1	38.6	34.7	36.6						
Current use, %	41.7	41.1	42.2	39.0	44.5	41.6						
Past use, %	22.4	22.4	20.8	22.5	20.8	21.8						
Aspirin/NSAID use, %	39.5	41	38.7	38.5	37.2	38.9	53.6	51.7	53.5	51.6	51.2	52.3
*Comorbidities*												
Hypertension, %	28.1	32.1	28.2	28.5	24.2	28.1	26.2	23.9	24.8	26.0	25.7	25.5
Hypercholesterolemia, %	39.3	40.9	39.1	36.7	32.0	37.6	33.2	33.5	34.8	35.9	38.2	34.9
*Contextual environment*												
NDVI-270 (cumulative updated average)	0.32 (0.09)	0.32 (0.08)	0.33 (0.08)	0.33 (0.08)	0.34 (0.09)	0.33 (0.09)	0.27 (0.11)	0.26 (0.1)	0.27 (0.09)	0.27 (0.09)	0.29 (0.1)	0.27 (0.10)
NDVI-270 (season of blood draw)	0.27 (0.21)	0.26 (0.2)	0.29 (0.2)	0.32 (0.2)	0.35 (0.19)	0.30 (0.20)	0.32 (0.16)	0.32 (0.17)	0.33 (0.16)	0.32 (0.16)	0.34 (0.16)	0.33 (0.17)
Air pollution (PM_2.5_) (μg/m^3^)	15.41 (4.21)	17.04 (3.81)	16.95 (3.71)	16.97 (3.50)	16.82 (3.41)	16.62 (3.80)	11.08 (3.32)	12.15 (3.13)	12.24 (3.05)	12.49 (2.98)	13.01 (2.72)	12.18 (3.15)
*Census tract SES*												
nSES z-score	−4.61 (1.84)	−2.11 (0.46)	−0.46 (0.50)	1.55 (0.71)	5.66 (2.50)	0.00 (3.77)	−4.52 (1.54)	−2.25 (0.44)	−0.64 (0.51)	1.52 (0.74)	5.84 (2.42)	0.00 (3.83)
Population density (100 people/mi^2^)	2.59 (4.05)	4.01 (5.70)	4.60 (4.86)	5.91 (8.13)	7.07 (17.23)	4.83 (9.30)	2.17 (3.58)	3.35 (4.93)	4.43 (5.34)	5.83 (7.18)	8.83 (1.99)	4.85 (19.95)
Income-ICE	0.00 (0.36)	0.28 (0.34)	0.52 (0.30)	0.69 (0.24)	0.83 (0.17)	0.46 (0.42)	−0.21 (0.37)	0.03 (0.4)	0.32 (0.39)	0.57 (0.36)	0.75 (0.29)	0.3 (0.51)
Joint Race/Income-ICE	0.44 (0.21)	0.58 (0.21)	0.69 (0.21)	0.76 (0.19)	0.82 (0.15)	0.66 (0.24)	0.3 (0.27)	0.44 (0.26)	0.58 (0.26)	0.71 (0.22)	0.79 (0.18)	0.57 (0.3)
*Inflammatory markers*												
Adiponectin, ng/L	10.59 (4.63)	10.75 (4.67)	10.75 (4.59)	10.9 (4.5)	11.22 (4.65)	10.85 (4.59)	6.42 (3.31)	6.62 (3.44)	6.68 (3.30)	6.33 (3.06)	6.97 (3.83)	6.59 (3.44)
C-reactive Protein, mg/L	3.31 (4.24)	3.15 (3.9)	3.03 (4)	3.07 (4.21)	2.75 (3.62)	3.08 (4.08)	1.75 (1.94)	1.81 (2.00)	1.70 (2.06)	1.73 (1.99)	1.64 (1.95)	1.72 (1.99)
IL-6, pg/mL	1.66 (1.43)	1.63 (1.53)	1.55 (1.33)	1.55 (1.38)	1.48 (1.47)	1.57 (1.40)	1.32 (0.89)	1.32 (0.83)	1.39 (0.95)	1.35 (0.94)	1.25 (0.86)	1.33 (0.92)
sTNFR-2, pg/L	2.72 (0.76)	2.68 (0.76)	2.65 (0.72)	2.61 (0.7)	2.53 (0.67)	2.64 (0.73)	2.66 (0.66)	2.61 (0.70)	2.60 (0.67)	2.61 (0.77)	2.54 (0.68)	2.61 (0.71)
Inflammation Score	0.56 (2.6)	0.2 (2.74)	0.05 (2.58)	−0.04 (2.61)	−0.38 (2.62)	0.08 (2.65)	0.13 (2.52)	0.02 (2.39)	−0.03 (2.44)	0.02 (2.67)	−0.45 (2.46)	−0.07 (2.5)

Abbreviations: MET: metabolic equivalent task units; ICE: Index of Concentration at Extremes; NDVI: Normalized Difference Vegetation Index; nSES: Neighborhood Socioeconomic Status; PM_2.5_: Particulate Matter ≤ 2.5 µm diameter, IL-6: Interleukin-6; sTNFR-2: soluble Tumor Necrosis Factor Receptor-2.

Values are means(SD) or medians(Q25, Q75) for continuous variables; percentages or ns or both for categorical variables, and are standardized to the age distribution of the study population.

Values of polytomous variables may not sum to 100% due to rounding.

* Value is not age adjusted
