# Supplementary material for: Impact of neighborhood socioeconomic status, income segregation, and greenness on blood biomarkers of inflammation
Source: Environ Int. Author manuscript; Available in PMC 2022 Apr 6. (PMC8985077; doi:10.1016/j.envint.2022.107164)
Supplement: Supp.materials [file NIHMS1786670-supplement-Supp_materials.pdf]

Supplementary Tables and Figures

Impact of neighborhood socioeconomic status, income segregation, and greenness on blood biomarkers of inflammation

Authors: Hari S. Iyer, Jaime E. Hart, Peter James, Elise G. Elliott, Nicole V. DeVille, Michelle D. Holmes, Immaculata De Vivo, Lorelei A. Mucci, Francine Laden, Timothy R. Rebbeck

Table of Contents

Supplementary Figure 1. Distribution of 1990 census tract-level median income, median home value, percent poverty, and percent White in the HPFS, NHS, and full United States

Supplementary Figure 2. Matrix of Spearman Correlations between neighborhood factors, individual demographic and lifestyle factors, and inflammatory blood biomarkers among women and men in the Nurses' Health Study and Health Professionals Follow-up Study

Supplementary Table 1. Summary statistics for inflammatory marker measures by laboratory batch

Supplementary Table 2. Associations between neighborhood contextual factors and inflammatory blood biomarkers from linear regression models among women and men in the Nurses' Health Study and Health Professionals Follow-up Study

Supplementary Table 3. Associations between neighborhood contextual factors, Interleukin-6, and inflammation score from linear regression models following sensitivity analysis among women and men in the Nurses' Health Study and Health Professionals Follow-up Study

Supplementary Table 4. Associations between neighborhood contextual measures and inflammatory biomarkers in the Health Professionals Follow-up Study stratified by address type (home vs work)

Supplementary Table 5. Associations between NDVI (270m) within two years of blood draw and inflammatory markers by census region

Supplementary Figure 1. Distribution of 1990 census tract-level median income, median home value, percent poverty, and percent White in the HPFS, NHS, and full United States

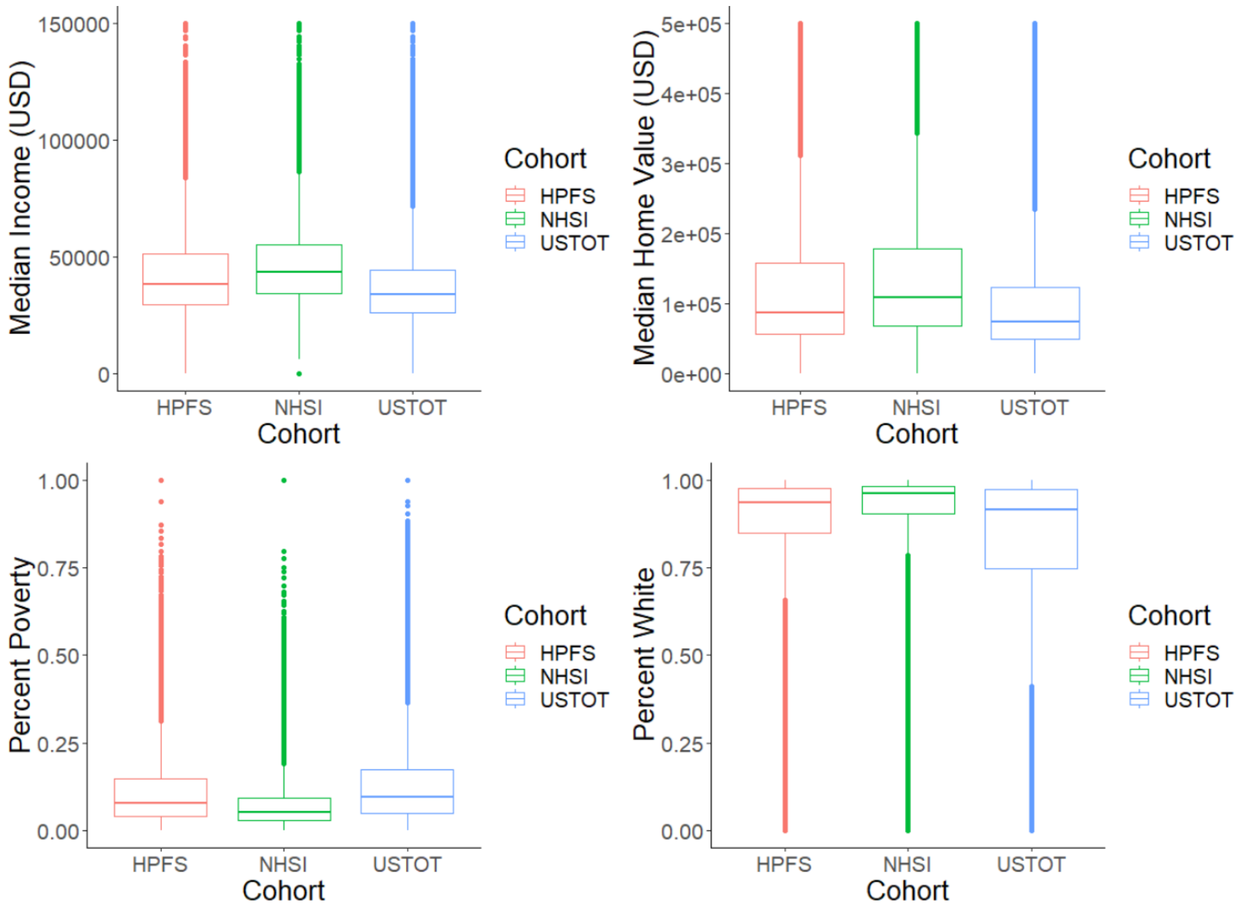

Abbreviations: NHS: Nurses' Health Study; HPFS: Health Professionals Follow-up Study, USTOT: Total US.

Supplementary Figure 2. Matrix of Spearman Correlations between neighborhood factors, individual demographic and lifestyle factors, and inflammatory blood biomarkers among women and men in the Nurses' Health Study and Health Professionals Follow-up Study

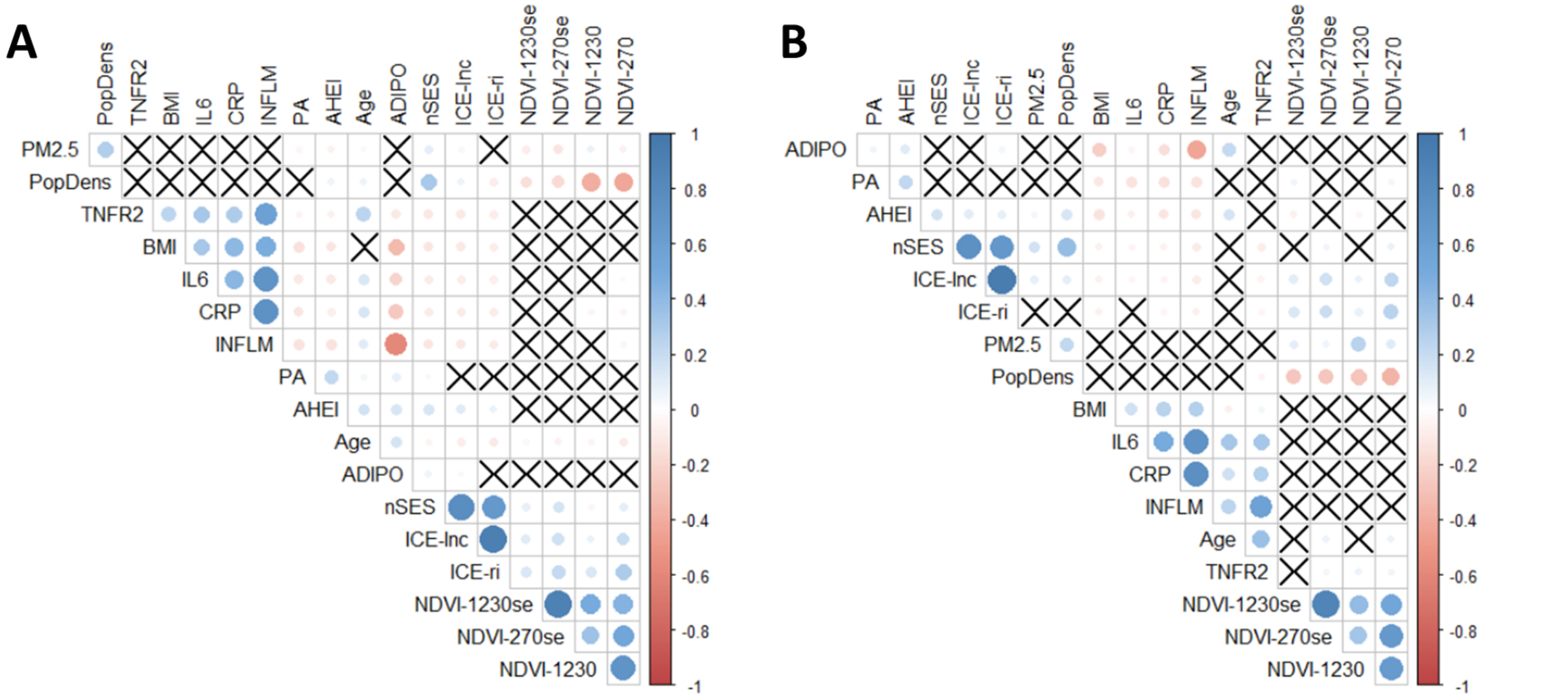

Panel A: women in NHS, Panel B: men in HPFS. Abbreviations: ADIPO: Adiponectin; AHEI: Alternative Health Eating Index; BMI: Body Mass Index; CRP: C-Reactive Protein; IL-6: Interleukin-6; TNFR-2: soluble tumor necrosis factor receptor-2; INFLM: inflammation score; nSES: Neighborhood Socioeconomic Status; ICE-Inc: Index of Concentration at Extremes-Income; ICE-RI: Index of Concentration at Extremes-Race/Income; NDVI: Normalized Difference Vegetation Index (NHS: 1986-1990, HPFS: 1990-1994), NDVI-1230se: 1230m seasonal Normalized Difference Vegetation Index; NDVI-270se: 270m seasonal Normalized Difference Vegetation Index; PM2.5: Particulate Matter, 2.5 microns or smaller in size; NHS: Nurses' Health Study; HPFS: Health Professionals Follow-up Study; PA: physical activity. Colors indicate positive (blue) and negative (red) correlations; X indicates lack of statistically significant correlation.

Supplementary Table 1. Summary statistics for inflammatory marker measures by laboratory batch

	N	Mean	Std Dev	Quartile 1	Median	Quartile 3	Minimum	Maximum
**NHS**								
**Adiponectin (ng/mL)**								
Lab 1	361	6763.65	4512.74	3718	5628	7978	1326	31740
Lab 2	1418	11411.29	3203.75	9180	11580	13700	1600	21350
Lab 3	1303	19982.04	4401.97	17510	20640	23120	2800	29750
Lab 4	574	9094.8	3944.8	6343.8	8448.8	11074	1567.6	28926.3
Lab 5	366	12725.32	6376.37	8057.1	11977	16465.4	1604.4	43930.3
Lab 6	529	9357.83	4094.84	6378.2	8655.4	11337.6	2644.6	33090.7
Lab 7	2017	15848.2	7541.65	9890	15130	20720	50	42130
Lab 8	788	8891.53	4024.83	5914.6	8345.5	11109.95	1803.8	27090
Lab 9	107	14403.41	6273.42	9756.6	13558.6	19814.9	3764	29832.4
Lab 10	1058	18506.75	7503.39	12950	17760	23440	3260	44020
Lab 11	303	8600.12	4784.73	5335.9	7789.9	10740.6	1688.3	39579.9
Lab 12	220	8354.36	4023.79	5381	7801.4	10534.35	1411.7	26502.6
Lab 13	677	8789.68	4653.36	5503.8	8007.9	10789.3	1446.8	37676.6
Lab 14	168	8445.84	3786.46	5502.35	7846.7	11017.5	1532	20774.1
Lab 15	97	8737.06	4111.83	5582.8	8298	11689.1	2006.6	22602.7
Lab 16	458	7517.84	3858.01	4836.4	6717.6	9333.7	1186	37131.3
Lab 17	106	15509.58	7633.01	9988	13872	19404	4318	41450
Lab 18	197	16418.57	7428.31	11575	15022	19507	3428	42146
**C-Reactive Protein (mg/L)**								
Lab 1	3	9.53	14.63	0.3	1.9	26.4	0.3	26.4
Lab 2	3	8.03	8.84	2.2	3.7	18.2	2.2	18.2
Lab 3	20	5.01	5.86	0.97	3.11	6.03	0.09	22.73
Lab 4	1024	7.09	6.64	2.11	4.79	9.78	0.38	30.45
Lab 5	247	2.44	3.47	0.46	1.32	2.88	0.08	27.78
Lab 6	1584	2.98	3.85	0.74	1.73	3.62	0.04	27.74
Lab 7	598	2.36	3.48	0.46	1.25	2.89	0.01	28.43
Lab 8	215	4.64	4.7	1.14	3.36	6.6	0.28	27.6
Lab 9	430	2.88	4.26	0.68	1.56	3.29	0.07	28.9
Lab 10	573	2.92	3.87	0.65	1.66	3.73	0.05	30.1
Lab 11	796	2.8	3.69	0.72	1.64	3.36	0.03	27.6
Lab 12	480	2.41	3.35	0.57	1.33	2.94	0.04	28.06
Lab 13	1073	2.7	3.74	0.66	1.53	3.4	0.02	29.38

	N	Mean	Std Dev	Quartile 1	Median	Quartile 3	Minimum	Maximum
Lab 14	64	3.6	2.87	1.35	2.48	5.38	0.31	11.4
Lab 15	137	2.9	4.39	0.72	1.4	3.11	0.08	30.34
Lab 16	126	2.51	3.57	0.69	1.25	2.71	0.04	22.22
Lab 17	317	2.61	3.23	0.62	1.54	3.48	0.04	25.35
Lab 18	787	3.36	4.02	0.85	2.02	4.34	0.04	29.27
Lab 19	221	2.43	2.8	0.56	1.44	3.45	0.02	19.69
Lab 20	603	3.49	5.01	0.87	1.99	3.92	0.03	30.61
Lab 21	148	2.66	3.6	0.47	1.44	3.16	0.1	24.11
Lab 22	33	2.35	2.34	0.51	1.77	3.23	0.14	9.84
Lab 23	480	2.57	3.75	0.56	1.28	2.98	0.07	27.84
Lab 24	428	2.77	3.19	0.81	1.76	3.69	0.04	27.34
Lab 25	82	1.78	1.42	0.75	1.35	2.59	0.09	7.18
Lab 26	458	2.93	3.86	0.74	1.68	3.57	0.06	28.91
**Interleukin-6 (pg/mL)**								
Lab 1	1276	2.5	1.72	1.43	2.07	3.06	0.03	12.47
Lab 2	427	2.29	1.74	1.21	1.74	2.76	0.41	12.4
Lab 3	199	2.1	1.57	1.15	1.64	2.58	0.34	10.67
Lab 4	260	1.56	1.46	0.81	1.15	1.77	0.3	12.3
Lab 5	594	1.26	1.35	0.63	0.9	1.35	0.31	12.35
Lab 6	799	1.68	1.57	0.83	1.21	1.89	0.23	11.17
Lab 7	507	1.61	1.44	0.74	1.14	1.92	0.21	11.52
Lab 8	1073	1.46	1.42	0.65	1.01	1.67	0.12	11.85
Lab 9	141	1.27	1.55	0.54	0.82	1.22	0.21	9.02
Lab 10	129	1.7	1.86	0.81	1.14	1.86	0.39	12.31
Lab 11	323	1.35	1.18	0.73	1	1.58	0.29	11.91
Lab 12	222	1.23	1.31	0.58	0.89	1.3	0.22	12.45
Lab 13	487	1.86	1.59	0.88	1.36	2.14	0.22	12.37
Lab 14	68	1.11	0.73	0.62	0.92	1.37	0.38	4.15
Lab 15	82	1.1	0.62	0.7	0.96	1.36	0.36	3.43
Lab 16	458	1.43	1.32	0.73	1.08	1.56	0.32	10.2
**soluble Tumor Necrosis Factor Receptor-2 (pg/L)**								
Lab 1	1274	2597.03	827.48	2086.17	2502.51	3018.63	82.34	5945.77
Lab 2	398	2475	705.86	2004.4	2350.55	2803.9	748.2	5640.2
Lab 3	186	2491.48	740.72	1965.7	2368.35	2905.1	1083.9	5436.3
Lab 4	845	2548.32	718.3	2044.86	2410.72	2968.99	939.35	6012.4

	N	Mean	Std Dev	Quartile 1	Median	Quartile 3	Minimum	Maximum
Lab 5	656	3124.24	726.57	2601.9	3075.7	3567.7	638.1	5942.6
Lab 6	254	2113.46	593.37	1745	1995	2378.6	1165.7	5527.6
Lab 7	587	2797.62	789.54	2286	2699.6	3218.5	1140.9	5706.5
Lab 8	791	2634.86	672.12	2127.9	2546.6	3007.5	1242.8	5609.6
Lab 9	460	2573.67	624.28	2169.15	2524.9	2884.6	1001.3	5326.5
Lab 10	1063	2369.17	658.89	1934.3	2238.5	2689	1070.8	5588.6
Lab 11	136	3119.76	779.51	2628.45	3091	3480.2	1356.5	5824.8
Lab 12	321	2672.74	682.28	2201	2576.1	3059.6	911.6	5349.7
Lab 13	214	2895.16	709.91	2415.4	2778.85	3279.3	1535	5374.5
Lab 14	468	2787.17	729.12	2287.15	2662.15	3120.15	1148.6	5898.1
Lab 15	68	2770.42	703.62	2214.6	2654.8	3155	1767.9	4689.1
Lab 16	494	2907.71	803.37	2328.8	2800.75	3357.5	1252.4	6080.8
Lab 17	78	2269.53	637.55	1811.3	2109.2	2519.6	1227.7	5140
Lab 18	445	2344.79	563.94	1948.7	2256.2	2621.2	1221.1	5716.4
**HPFS**								
**Adiponectin (ng/mL)**								
Lab 1	829	14712.9	6409.85	10025	13880	18525	1405	32750
Lab 2	11	7400.91	2139.69	5360	7490	9250	3380	10430
Lab 3	213	5801.2	3284.9	3879.5	5141	7102.2	1502.7	28802.6
Lab 4	880	5855.54	2883.78	3886.9	5127.25	7147.95	1366.1	26358.5
Lab 5	176	6395.26	3427.95	4105.1	5720.3	8262.1	1142.7	20850.5
Lab 6	350	5688.95	2862.98	3740.7	5017.1	6918.8	1385.7	20200.5
Lab 7	760	6062.47	3509.61	3527.25	5297	7517.6	1150.8	30829.9
Lab 8	464	6023.15	3393.43	3702.8	5274.65	7449.25	1537.9	31880.7
Lab 9	308	5160.36	2534.5	3334.1	4649.4	6439.35	1532.9	17518.2
Lab 10	23	4509.25	1919.04	3329.7	4186.5	5157.8	2105	10591.8
Lab 11	65	9764.02	4449.63	6456	9407	12475	1729	19783
Lab 12	127	12016.66	6761.4	6277	10916	15850	1879	32833
Lab 13	153	14671.85	6957.31	8987	13540	18991	3201	32565
**C-Reactive Protein (mg/L)**								
Lab 1	799	2.64	2.77	0.89	1.76	3.48	0.04	16.73
Lab 2	669	2.03	2.47	0.56	1.13	2.44	0.02	14.9
Lab 3	239	1.83	1.83	0.61	1.15	2.42	0.15	9.55
Lab 4	473	1.8	1.92	0.58	1.2	2.3	0.09	14.96
Lab 5	228	1.52	1.57	0.54	0.92	1.89	0.12	9.07

	N	Mean	Std Dev	Quartile 1	Median	Quartile 3	Minimum	Maximum
Lab 6	490	1.48	1.82	0.46	0.88	1.69	0.07	15.96
Lab 7	194	1.75	2.07	0.47	1.09	2.11	0.09	15.58
Lab 8	58	1.2	1.23	0.43	0.75	1.53	0.16	7.22
Lab 9	861	1.62	1.79	0.46	0.97	2.05	0.05	14.21
Lab 10	278	1.76	2.27	0.59	1.02	1.89	0.04	15.79
Lab 11	154	1.72	2.14	0.5	0.97	2.1	0.07	15.25
Lab 12	295	1.76	1.88	0.56	1.09	2.21	0.07	9.51
Lab 13	395	1.75	1.84	0.56	1.21	2.28	0.04	16.45
Lab 14	486	1.82	2.09	0.55	1.03	2.24	0.06	13.6
Lab 15	299	1.52	1.92	0.42	0.83	1.84	0.02	12.27
Lab 16	22	1.5	1.49	0.64	0.92	1.48	0.2	5.37
Lab 17	136	1.86	2.29	0.48	0.96	2.2	0.1	11.53
**Interleukin-6 (pg/mL)**								
Lab 1	376	1.33	0.97	0.72	1.06	1.61	0.15	5.99
Lab 2	272	1.59	1.11	0.86	1.23	1.99	0.25	6.09
Lab 3	227	1.32	0.86	0.77	1.06	1.61	0.32	5.13
Lab 4	72	1.44	1.06	0.73	1.02	1.86	0.39	4.8
Lab 5	852	1.4	0.96	0.77	1.1	1.66	0.26	6.3
Lab 6	739	1.73	1.11	0.94	1.4	2.13	0.29	6.37
Lab 7	337	1.12	0.83	0.64	0.88	1.28	0.27	6.45
Lab 8	27	1.22	0.8	0.7	0.95	1.74	0.37	4.02
Lab 9	136	1.25	0.9	0.66	0.9	1.55	0.29	5.87
**soluble Tumor Necrosis Factor-2 (pg/mL)**								
Lab 1	829	2957.61	855.43	2322.3	2816	3430	1330.2	6034.3
Lab 2	269	2453.88	645.82	2020	2389.2	2731.1	1225.6	5893.4
Lab 3	218	2566.22	682.48	2110.6	2431.55	2903.3	1251	5666.5
Lab 4	881	2602.13	679.57	2135.2	2509.9	2958.2	732.5	6079.2
Lab 5	760	2875.12	785.12	2330.25	2711.15	3273.55	1315.6	5971.6
Lab 6	312	2637.52	714.43	2150.8	2516.65	2966.2	1391.2	5654.9
Lab 7	324	2495.35	587.85	2055.8	2428.1	2814.85	1440.6	4714.1
Lab 8	26	2609.34	696.39	2096.4	2414.1	3150.8	1669.2	4222.5

Supplementary Table 2. Associations between neighborhood contextual factors and inflammatory blood biomarkers from linear regression models among women and men in the Nurses' Health Study and Health Professionals Follow-up Study

Biomarker	n	Contextual Factor	Cont	Q1	Q2	Q3	Q4	Q5	P_{trend}
**Nurses' Health Study (women)**									
**ADIPO**	9837	NDVI-270m	1.07 (-0.35, 2.49)	Ref	-1.02 (-3.75, 1.78)	1.24 (-1.67, 4.23)	2.55 (-0.52, 5.72)	1.67 (-1.43, 4.88)	0.0872
	9837	NDVI-1230m	0.62 (-0.68, 1.95)	Ref	0.24 (-2.55, 3.11)	0.69 (-2.15, 3.6)	2.18 (-0.77, 5.23)	0.88 (-2.07, 3.93)	0.3172
	9837	NDVI-270s	-1.13 (-2.75, 0.51)	Ref	-1.52 (-4.37, 1.41)	-0.7 (-3.5, 2.18)	-3.04 (-5.80, -0.21)	-1.15 (-4.16, 1.94)	0.238
	9837	nSES	2.39 (1.25, 3.53)	Ref	2.06 (-0.71, 4.90)	2.16 (-0.62, 5.01)	3.81 (0.97, 6.73)	5.23 (2.3, 8.25)	0.0003
	9835	ICE-income	1.92 (0.62, 3.23)	Ref	-0.50 (-3.15, 2.23)	1.34 (-1.38, 4.13)	2.93 (0.13, 5.81)	2.97 (0.15, 5.87)	0.0077
	9835	ICE-race/income	2.13 (0.93, 3.34)	Ref	1.15 (-1.57, 3.94)	0.68 (-2.04, 3.48)	3.95 (1.11, 6.87)	4.83 (1.95, 7.81)	0.0005
**CRP**	9625	NDVI-270m	-4.36 (-7.74, -0.87)	Ref	-0.45 (-7.32, 6.92)	-0.45 (-7.61, 7.26)	-3.17 (-10.41, 4.66)	-8.44 (-15.48, -0.81)	0.0333
	9625	NDVI-1230m	-2.24 (-5.48, 1.11)	Ref	3.75 (-3.50, 11.55)	0.78 (-6.34, 8.46)	2.02 (-5.38, 9.99)	-3.01 (-10.14, 4.67)	0.3344
	9625	NDVI-270s	1.21 (-2.99, 5.59)	Ref	-3.42 (-10.4, 4.11)	4.51 (-2.92, 12.49)	3.99 (-3.47, 12.02)	-1.29 (-8.81, 6.85)	0.5101
	9625	nSES	-8.38 (-10.99, -5.69)	Ref	-5.93 (-12.31, 0.92)	-9.65 (-15.80, -3.05)	-9.45 (-15.65, -2.80)	-18.87 (-24.56, -12.74)	<.0001
	9623	ICE-income	-6.98 (-9.99, -3.88)	Ref	-2.25 (-8.81, 4.78)	-8.11 (-14.31, -1.46)	-12.53 (-18.52, -6.12)	-9.50 (-15.73, -2.81)	<.0001
	9623	ICE-race/income	-6.08 (-8.92, -3.16)	Ref	-6.36 (-12.65, 0.38)	-5.52 (-11.98, 1.40)	-13.29 (-19.22, -6.93)	-13.14 (-19.19, -6.65)	<.0001
**IL6**	6622	NDVI-270m	-3.56 (-5.97, -1.08)	Ref	-1.09 (-5.96, 4.01)	-1.49 (-6.50, 3.80)	-5.09 (-10.06, 0.16)	-5.37 (-10.51, 0.06)	0.0236
	6622	NDVI-1230m	-2.02 (-4.29, 0.31)	Ref	-0.69 (-5.61, 4.49)	-1.92 (-6.82, 3.22)	-0.05 (-5.17, 5.35)	-4.60 (-9.52, 0.58)	0.1203
	6622	NDVI-270s	-3.9 (-6.66, -1.06)	Ref	-4.15 (-9.06, 1.03)	-2.97 (-7.82, 2.14)	-3.39 (-8.2, 1.68)	-8.74 (-13.58, -3.64)	0.0061
	6622	nSES	-5.19 (-7.07, -3.28)	Ref	-3.82 (-8.37, 0.95)	-6.84 (-11.27, -2.19)	-8.30 (-12.67, -3.72)	-11.45 (-15.79, -6.87)	<.0001
	6621	ICE-income	-4.96 (-7.09, -2.79)	Ref	-4.04 (-8.55, 0.70)	-8.73 (-13.04, -4.20)	-5.12 (-9.65, -0.36)	-9.65 (-13.99, -5.09)	<.0001
	6621	ICE-race/income	-3.86 (-5.87, -1.80)	Ref	-3.02 (-7.62, 1.81)	-7.60 (-12.01, -2.95)	-10.07 (-14.4, -5.51)	-8.01 (-12.47, -3.32)	<.0001
**TNFR2**	7683	NDVI-270m	-0.44 (-1.47, 0.59)	Ref	0.02 (-2.01, 2.10)	-0.64 (-2.73, 1.51)	-0.43 (-2.61, 1.79)	-0.19 (-2.43, 2.11)	0.7643
	7683	NDVI-1230m	-0.29 (-1.24, 0.68)	Ref	-0.95 (-2.97, 1.14)	-1.45 (-3.50, 0.63)	-1.70 (-3.80, 0.45)	-0.54 (-2.67, 1.64)	0.5381
	7683	NDVI-270s	1.36 (0.15, 2.58)	Ref	-0.99 (-3.10, 1.17)	-0.21 (-2.28, 1.92)	1.66 (-0.46, 3.83)	1.66 (-0.60, 3.97)	0.0301
	7683	nSES	-1.95 (-2.74, -1.14)	Ref	-1.63 (-3.56, 0.35)	-1.77 (-3.72, 0.21)	-2.80 (-4.74, -0.83)	-4.66 (-6.61, -2.66)	<.0001
	7681	ICE-income	-1.97 (-2.89, -1.05)	Ref	-2.00 (-3.90, -0.05)	-0.97 (-2.92, 1.03)	-3.11 (-5.04, -1.14)	-3.89 (-5.82, -1.92)	0.0002
	7681	ICE-race/income	-1.54 (-2.39, -0.67)	Ref	-1.34 (-3.28, 0.63)	-2.30 (-4.25, -0.31)	-2.58 (-4.53, -0.57)	-3.95 (-5.91, -1.95)	<.0001
**INFLM**	3897	NDVI-270m	-18.88 (-28.91, -7.44)	Ref	-6.78 (-28.47, 21.47)	-16.88 (-36.83, 9.36)	-20.08 (-39.75, 6.00)	-29.66 (-47.54, -5.69)	0.0131
	3897	NDVI-1230m	-11.73 (-21.85, -0.31)	Ref	-8.30 (-29.85, 19.85)	-12.51 (-33.04, 14.34)	-20.26 (-39.50, 5.10)	-24.06 (-42.44, 0.20)	0.0315
	3897	NDVI-270s	5.92 (-9.23, 23.6)	Ref	-17.95 (-37.62, 7.91)	1.79 (-22.13, 33.06)	11.20 (-15.07, 45.6)	-10.58 (-32.97, 19.29)	0.7852
	3897	nSES	-27.73 (-34.91, -19.76)	Ref	-33.11 (-48.19, -13.64)	-39.40 (-53.12, -21.66)	-44.73 (-57.18, -28.66)	-54.57 (-65.12, -40.81)	<.0001
	3896	ICE-income	-27.75 (-35.83, -18.66)	Ref	-25.02 (-41.70, -3.56)	-36.77 (-51.06, -18.32)	-35.15 (-49.88, -16.10)	-45.37 (-57.76, -29.33)	<.0001
	3896	ICE-race/income	-23.13 (-31.22, -14.07)	Ref	-22.29 (-39.57, -0.07)	-30.64 (-46.47, -10.11)	-45.00 (-57.63, -28.61)	-44.69 (-57.33, -28.32)	<.0001
**Health Professionals Follow-up Study (men)**									
**ADIPO**	3396	NDVI-270m	1.05 (-1.53, 3.70)	Ref	-0.87 (-5.77, 4.29)	5.01 (-0.24, 10.53)	0.52 (-4.63, 5.96)	1.84 (-3.53, 7.50)	0.47
	3396	NDVI-1230m	0.72 (-1.40, 2.90)	Ref	4.93 (-0.17, 10.29)	1.41 (-3.72, 6.82)	3.71 (-1.64, 9.33)	1.52 (-3.78, 7.10)	0.60

	3396 NDVI-270s	-0.01 (-2.38, 2.41)	Ref	1.19 (-3.82, 6.46)	3.37 (-1.65, 8.63)	3.17 (-1.94, 8.53)	-0.35 (-5.29, 4.85)	0.84
	3396 nSES	1.52 (-0.61, 3.70)	Ref	0.65 (-4.23, 5.79)	0.61 (-4.23, 5.70)	-1.83 (-6.66, 3.24)	2.99 (-2.28, 8.56)	0.43
	3395 ICE-income	2.62 (-0.11, 5.42)	Ref	3.67 (-1.33, 8.90)	3.77 (-1.24, 9.04)	4.29 (-0.79, 9.61)	3.89 (-1.20, 9.25)	0.11
	3395 ICE-race/income	2.65 (0.38, 4.98)	Ref	5.36 (0.21, 10.78)	6.50 (1.36, 11.91)	4.53 (-0.60, 9.92)	4.26 (-0.92, 9.69)	0.10
**CRP**	5244 NDVI-270m	0.44 (-3.65, 4.72)	Ref	5.07 (-3.13, 13.99)	-4.64 (-12.17, 3.55)	4.12 (-4.36, 13.36)	-0.29 (-8.58, 8.75)	0.92
	5244 NDVI-1230m	0.09 (-3.33, 3.63)	Ref	-0.63 (-8.36, 7.77)	1.25 (-6.91, 10.12)	-0.51 (-8.64, 8.35)	0.93 (-7.46, 10.09)	0.85
	5244 NDVI-270s	0.68 (-3.10, 4.61)	Ref	7.12 (-1.22, 16.18)	9.82 (1.28, 19.09)	3.34 (-4.78, 12.16)	3.22 (-4.89, 12.02)	0.65
	5244 nSES	-1.58 (-4.86, 1.82)	Ref	6.12 (-2.05, 14.96)	-3.24 (-10.65, 4.78)	1.12 (-6.80, 9.70)	-0.38 (-8.45, 8.42)	0.61
	5242 ICE-income	0.57 (-3.74, 5.06)	Ref	-1.1 (-8.63, 7.05)	-1.33 (-8.98, 6.95)	1.38 (-6.46, 9.88)	-0.71 (-8.52, 7.79)	0.92
	5242 ICE-race/income	1.44 (-2.26, 5.30)	Ref	0.98 (-6.82, 9.44)	3.59 (-4.44, 12.3)	-0.64 (-8.41, 7.78)	2.3 (-5.85, 11.14)	0.73
**IL6**	2751 NDVI-270m	0.25 (-3.07, 3.69)	Ref	8.92 (2.07, 16.23)	4.22 (-2.49, 11.38)	5.18 (-1.81, 12.67)	2.48 (-4.43, 9.89)	0.84
	2751 NDVI-1230m	-0.05 (-2.79, 2.76)	Ref	-2.32 (-8.43, 4.19)	-0.63 (-7.11, 6.31)	-3.34 (-9.78, 3.56)	-1.43 (-8.03, 5.64)	0.60
	2751 NDVI-270s	-4.07 (-7.03, -1.01)	Ref	-3.47 (-9.66, 3.16)	-1.61 (-7.75, 4.95)	-6.28 (-12.25, 0.08)	-7.55 (-13.52, -1.15)	0.016
	2751 nSES	-1.71 (-4.35, 1.01)	Ref	2.71 (-3.79, 9.64)	-1.22 (-7.48, 5.44)	-2.05 (-8.26, 4.59)	-2.38 (-8.92, 4.62)	0.24
	2749 ICE-income	-0.48 (-3.96, 3.12)	Ref	-0.71 (-6.94, 5.95)	-3.08 (-9.19, 3.45)	0.05 (-6.34, 6.88)	-2.36 (-8.62, 4.32)	0.57
	2749 ICE-race/income	0.01 (-2.97, 3.08)	Ref	3.95 (-2.68, 11.03)	0.47 (-5.83, 7.20)	4.16 (-2.52, 11.29)	-0.08 (-6.54, 6.83)	0.89
**TNFR2**	2985 NDVI-270m	0.13 (-1.29, 1.56)	Ref	-0.45 (-3.14, 2.32)	0.6 (-2.19, 3.47)	-0.31 (-3.16, 2.63)	0.38 (-2.56, 3.41)	0.80
	2985 NDVI-1230m	1.14 (-0.03, 2.32)	Ref	1.12 (-1.62, 3.93)	1.6 (-1.22, 4.50)	2.06 (-0.86, 5.07)	2.70 (-0.23, 5.71)	0.060
	2985 NDVI-270s	-0.28 (-1.57, 1.03)	Ref	-2.47 (-5.15, 0.29)	-2.72 (-5.33, -0.04)	-2.07 (-4.72, 0.65)	-1.17 (-3.87, 1.60)	0.45
	2985 nSES	-2.18 (-3.30, -1.04)	Ref	-3.59 (-6.17, -0.94)	-4.23 (-6.78, -1.60)	-4.66 (-7.26, -1.99)	-5.57 (-8.25, -2.81)	0.0004
	2983 ICE-income	-1.68 (-3.13, -0.20)	Ref	0.02 (-2.64, 2.76)	-2.25 (-4.86, 0.41)	-1.88 (-4.55, 0.86)	-2.07 (-4.75, 0.70)	0.045
	2983 ICE-race/income	-0.83 (-2.06, 0.42)	Ref	-0.80 (-3.48, 1.96)	-1.77 (-4.38, 0.90)	-1.91 (-4.61, 0.85)	-2.71 (-5.40, 0.04)	0.037
**INFLM**	2040 NDVI-270m	-0.86 (-15.74, 16.66)	Ref	40.68 (2.89, 92.34)	-2.22 (-29.59, 35.80)	20.27 (-13.84, 67.88)	1.03 (-28.05, 41.84)	0.74
	2040 NDVI-1230m	-2.62 (-14.90, 11.40)	Ref	-14.25 (-37.36, 17.39)	-15.91 (-39.63, 17.12)	-8.48 (-34.69, 28.25)	-10.99 (-36.40, 24.57)	0.55
	2040 NDVI-270s	-3.50 (-17.08, 12.31)	Ref	-4.61 (-31.03, 31.92)	6.98 (-21.82, 46.37)	-16.63 (-39.47, 14.82)	-4.74 (-31.22, 31.93)	0.56
	2040 nSES	-21.21 (-30.95, -10.09)	Ref	-16.91 (-39.72, 14.53)	-27.65 (-47.25, -0.78)	-23.45 (-44.53, 5.65)	-39.46 (-56.76, -15.24)	0.0053
	2039 ICE-income	-16.41 (-29.72, -0.56)	Ref	-19.24 (-41.15, 10.83)	-25.28 (-45.51, 2.47)	-21.40 (-43.25, 8.87)	-27.23 (-47.25, 0.38)	0.056
	2039 ICE-race/income	-13.81 (-25.61, -0.14)	Ref	-15.84 (-39.08, 16.25)	-17.68 (-39.85, 12.66)	-16.05 (-39.33, 16.17)	-26.60 (-47.05, 1.73)	0.091

Abbreviations: ADIPO: Adiponectin; CRP: C-Reactive Protein; IL-6: Interleukin-6; TNFR-2: soluble tumor necrosis factor receptor-2; INFLM: inflammation score; nSES: Neigborhood Socioeconomic Status; ICE-Inc: Index of Concentration at Extremes-Income; ICE-RI: Index of Concentration at Extremes-Race/Income; NDVI: Normalized Difference Vegetation Index (NHS: 1986-1990, HPFS: 1990-1994), NDVI-270se: 270m seasonal Normalized Difference Vegetation Index. NHS: Nurses' Health Study; HPFS: Health Professionals Follow-up Study. All variables are scaled to one-interquartile range increase except ICE-measures which are scaled to standard deviation. Multiple linear regression models for inflammatory markers adjusted for age, fasting status, smoking, hypertension, hypercholesterolemia, body mass index, census region, population density, case status, air pollution ($PM_{2.5}$), and use of anti-inflammatory medication. For NHS, models further adjusted for postmenopausal hormone use. Models for association between NDVI and inflammatory biomarkers were adjusted for nSES.

Supplementary Table 3. Associations between neighborhood contextual factors, Interleukin-6, and inflammation score from linear regression models following sensitivity analysis among women and men in the Nurses' Health Study and Health Professionals Follow-up Study

buff	Sensitivity	n	Cont	Q1	Q2	Q3	Q4	Q5	Trend
**INFLM**									
**Nurses' Health Study**									
NDVI270	Conf	3897	-18.88 (-28.91, -7.44)	Ref	-6.78 (-28.47, 21.47)	-16.88 (-36.83, 9.36)	-20.08 (-39.75, 6.00)	-29.66 (-47.54, -5.69)	0.0131
	Med	3897	-16.44 (-26.65, -4.80)	Ref	-2.95 (-25.28, 26.06)	-12.60 (-33.35, 14.59)	-13.48 (-34.56, 14.39)	-24.15 (-43.23, 1.34)	0.051
	Controls	2326	-20.12 (-32.29, -5.76)	Ref	-12.72 (-37.31, 21.51)	2.53 (-27.01, 44.02)	-22.56 (-45.67, 10.37)	-28.67 (-50.62, 3.05)	0.07
	Nomov	3832	-19.43 (-29.47, -7.96)	Ref	-6.13 (-28.14, 22.62)	-16.49 (-36.69, 10.15)	-19.87 (-39.74, 6.55)	-30.98 (-48.68, -7.19)	0.01
ICE-inc	Conf	3896	-27.75 (-35.83, -18.66)	Ref	-25.02 (-41.70, -3.56)	-36.77 (-51.06, -18.32)	-35.15 (-49.88, -16.10)	-45.37 (-57.76, -29.33)	<.0001
	Med	3896	-23.37 (-31.88, -13.80)	Ref	-20.90 (-38.29, 1.41)	-31.17 (-46.58, -11.31)	-29.53 (-45.37, -9.11)	-38.88 (-52.65, -21.12)	<.0001
	Controls	2325	-27.73 (-37.69, -16.16)	Ref	-22.77 (-43.58, 5.71)	-32.84 (-51.31, -7.36)	-37.07 (-54.47, -13.00)	-40.74 (-56.83, -18.65)	0.0003
	Nomov	3831	-27.91 (-36.03, -18.76)	Ref	-24.35 (-41.29, -2.50)	-37.46 (-51.68, -19.05)	-35.65 (-50.35, -16.59)	-45.89 (-58.27, -29.83)	<.0001
nSES	Conf	3897	-27.73 (-34.91, -19.76)	Ref	-33.11 (-48.19, -13.64)	-39.40 (-53.12, -21.66)	-44.73 (-57.18, -28.66)	-54.57 (-65.12, -40.81)	<.0001
	Med	3897	-23.75 (-31.28, -15.39)	Ref	-31.83 (-47.03, -12.26)	-33.17 (-48.16, -13.83)	-39.94 (-53.36, -22.66)	-48.4 (-60.32, -32.89)	<.0001
	Controls	2326	-23.04 (-32.27, -12.56)	Ref	-36.05 (-53.55, -11.97)	-49.64 (-63.60, -30.31)	-43.22 (-58.78, -21.78)	-51.78 (-65.19, -33.18)	<.0001
	Nomov	3832	-28.03 (-35.25, -20.00)	Ref	-33.32 (-48.45, -13.74)	-40.12 (-53.76, -22.45)	-44.98 (-57.48, -28.79)	-54.94 (-65.51, -41.13)	<.0001
**Health Professionals Follow-up Study**									
NDVI270	Conf	2040	-0.86 (-15.74, 16.66)	Ref	40.68 (2.89, 92.34)	-2.22 (-29.59, 35.80)	20.27 (-13.84, 67.88)	1.03 (-28.05, 41.84)	0.74
	Med	2040	-2.39 (-16.96, 14.74)	Ref	36.56 (0.15, 86.21)	-4.02 (-30.71, 32.95)	16.52 (-16.28, 62.16)	-2.95 (-30.71, 35.95)	0.59
	Controls	1211	-2.12 (-20.85, 21.05)	Ref	74.89 (16.18, 163.27)	-4.31 (-37.99, 47.65)	21.18 (-21.13, 86.19)	10.85 (-29.00, 73.07)	0.75
	Nomov	1914	0.71 (-14.89, 19.17)	Ref	43.35 (3.85, 97.84)	5.57 (-24.86, 48.32)	21.56 (-13.97, 71.74)	7.48 (-24.3, 52.59)	0.97
ICE-inc	Conf	2039	-16.41 (-29.72, -0.56)	Ref	-19.24 (-41.15, 10.83)	-25.28 (-45.51, 2.47)	-21.40 (-43.25, 8.87)	-27.23 (-47.25, 0.38)	0.056
	Med	2039	-15.14 (-28.61, 0.87)	Ref	-18.34 (-40.32, 11.74)	-23.34 (-43.97, 4.89)	-20.70 (-42.66, 9.68)	-25.64 (-46.00, 2.38)	0.073
	Controls	1210	-2.60 (-22.07, 21.74)	Ref	-20.85 (-47.52, 19.36)	-17.93 (-45.3, 23.13)	-7.30 (-39.24, 41.45)	-5.85 (-37.38, 41.55)	0.93
	Nomov	1913	-15.95 (-29.67, 0.44)	Ref	-17.69 (-40.51, 13.86)	-22.98 (-44.4, 6.69)	-23.64 (-45.44, 6.88)	-25.26 (-46.28, 4.01)	0.067
nSES	Conf	2040	-21.21 (-30.95, -10.09)	Ref	-16.91 (-39.72, 14.53)	-27.65 (-47.25, -0.78)	-23.45 (-44.53, 5.65)	-39.46 (-56.76, -15.24)	0.0053
	Med	2040	-18.45 (-28.52, -6.95)	Ref	-12.39 (-36.29, 20.49)	-23.41 (-44.03, 4.81)	-19.32 (-41.42, 11.10)	-33.78 (-52.68, -7.34)	0.019
	Controls	1211	-12.49 (-25.93, 3.41)	Ref	-4.03 (-36.82, 45.79)	-25.07 (-50.02, 12.34)	-7.17 (-39.10, 41.48)	-10.15 (-41.4, 37.75)	0.74
	Nomov	1914	-20.78 (-30.82, -9.29)	Ref	-13.83 (-38.04, 19.85)	-26.88 (-47.10, 1.08)	-23.87 (-45.46, 6.27)	-37.87 (-55.93, -12.40)	0.0072
**IL6**									
**Nurses' Health Study**									
NDVI270	Conf	6622	-3.56 (-5.97, -1.08)	Ref	-1.09 (-5.96, 4.01)	-1.49 (-6.50, 3.80)	-5.09 (-10.06, 0.16)	-5.37 (-10.51, 0.06)	0.0236
	Med	6622	-3.30 (-5.7, -0.85)	Ref	-0.47 (-5.32, 4.63)	-0.92 (-5.92, 4.35)	-4.33 (-9.31, 0.90)	-4.73 (-9.87, 0.68)	0.0411
	Controls	4247	-3.81 (-6.79, -0.73)	Ref	-2.37 (-8.30, 3.95)	-1.62 (-7.75, 4.93)	-5.96 (-12.01, 0.50)	-6.82 (-13.04, -0.14)	0.0297
	Nomov	6508	-3.65 (-6.08, -1.16)	Ref	-1.46 (-6.33, 3.68)	-1.72 (-6.76, 3.58)	-5.33 (-10.32, -0.06)	-5.72 (-10.87, -0.27)	0.0186
ICE-inc	Conf	6621	-4.96 (-7.09, -2.79)	Ref	-4.04 (-8.55, 0.70)	-8.73 (-13.04, -4.20)	-5.12 (-9.65, -0.36)	-9.65 (-13.99, -5.09)	<.0001
	Med	6621	-4.08 (-6.22, -1.90)	Ref	-3.25 (-7.77, 1.49)	-7.51 (-11.86, -2.96)	-3.72 (-8.30, 1.08)	-8.12 (-12.52, -3.51)	0.001
	Controls	4246	-5.14 (-7.79, -2.42)	Ref	-3.83 (-9.42, 2.10)	-9.05 (-14.38, -3.40)	-5.11 (-10.73, 0.86)	-9.77 (-15.09, -4.11)	0.0009
	Nomov	6507	-4.93 (-7.07, -2.74)	Ref	-3.82 (-8.38, 0.96)	-8.82 (-13.15, -4.26)	-5.53 (-10.08, -0.77)	-9.38 (-13.77, -4.77)	<.0001

nSES	Conf	6622	-5.19 (-7.07, -3.28)	Ref	-3.82 (-8.37, 0.95)	-6.84 (-11.27, -2.19)	-8.30 (-12.67, -3.72)	-11.45 (-15.79, -6.87)	<.0001
	Med	6622	-4.33 (-6.23, -2.40)	Ref	-3.50 (-8.03, 1.25)	-5.75 (-10.20, -1.06)	-7.03 (-11.44, -2.41)	-9.62 (-14.05, -4.96)	<.0001
	Controls	4247	-5.43 (-7.7, -3.10)	Ref	-3.74 (-9.34, 2.22)	-8.57 (-13.99, -2.82)	-7.47 (-12.93, -1.66)	-12.37 (-17.62, -6.78)	<.0001
	Nomov	6508	-5.26 (-7.14, -3.32)	Ref	-4.07 (-8.63, 0.73)	-6.95 (-11.40, -2.27)	-8.11 (-12.52, -3.49)	-11.93 (-16.28, -7.35)	<.0001
**Health Professionals Follow-up Study**									
NDVI270	Conf	2751	0.25 (-3.07, 3.69)	Ref	8.92 (2.07, 16.23)	4.22 (-2.49, 11.38)	5.18 (-1.81, 12.67)	2.48 (-4.43, 9.89)	0.84
	Med	2751	-0.21 (-3.50, 3.20)	Ref	8.32 (1.55, 15.52)	4.04 (-2.61, 11.14)	4.47 (-2.43, 11.86)	1.53 (-5.28, 8.84)	0.97
	Controls	1848	0.80 (-3.35, 5.15)	Ref	13.80 (4.96, 23.40)	8.24 (-0.38, 17.61)	9.71 (0.79, 19.43)	4.36 (-4.37, 13.89)	0.64
	Nomov	2589	0.59 (-2.86, 4.15)	Ref	8.94 (1.89, 16.47)	4.46 (-2.49, 11.88)	6.00 (-1.28, 13.83)	3.41 (-3.78, 11.14)	0.63
ICE-inc	Conf	2749	-0.48 (-3.96, 3.12)	Ref	-0.71 (-6.94, 5.95)	-3.08 (-9.19, 3.45)	0.05 (-6.34, 6.88)	-2.36 (-8.62, 4.32)	0.57
	Med	2749	-0.31 (-3.78, 3.29)	Ref	-0.63 (-6.83, 5.98)	-2.81 (-8.91, 3.70)	0.18 (-6.19, 6.98)	-2.17 (-8.41, 4.50)	0.61
	Controls	1779	0.37 (-3.91, 4.83)	Ref	0.13 (-7.60, 8.50)	-2.00 (-9.52, 6.14)	-0.47 (-8.27, 7.98)	1.27 (-6.57, 9.76)	0.91
	Nomov	2587	-0.56 (-4.12, 3.14)	Ref	-0.07 (-6.51, 6.80)	-2.38 (-8.72, 4.40)	-1.28 (-7.77, 5.66)	-1.66 (-8.12, 5.26)	0.53
nSES	Conf	2751	-1.71 (-4.35, 1.01)	Ref	2.71 (-3.79, 9.64)	-1.22 (-7.48, 5.44)	-2.05 (-8.26, 4.59)	-2.38 (-8.92, 4.62)	0.24
	Med	2751	-1.13 (-3.80, 1.59)	Ref	3.22 (-3.27, 10.14)	-0.21 (-6.49, 6.50)	-1.18 (-7.42, 5.49)	-1.08 (-7.71, 6.00)	0.41
	Controls	1781	-1.34 (-4.58, 2.02)	Ref	4.64 (-3.55, 13.53)	-0.87 (-8.55, 7.47)	-1.32 (-9.05, 7.06)	0.79 (-7.41, 9.74)	0.74
	Nomov	2589	-1.89 (-4.61, 0.90)	Ref	2.55 (-4.13, 9.71)	-2.2 (-8.54, 4.59)	-3.11 (-9.46, 3.69)	-2.53 (-9.21, 4.64)	0.22

Abbreviations: Conf: main models adjusted for variables described below. Med: mediation models adjusted for physical activity and diet quality. nSES: Neigborhood Socioeconomic Status; ICE-Inc: Index of Concentration at Extremes-Income; NDVI: Normalized Difference Vegetation Index (NHS: 1986-1990, HPFS: 1990-1994), NHS: Nurses' Health Study; HPFS: Health Professionals Follow-up Study. All variables are scaled to one-interquartile range increase except ICE-measures which are scaled to standard deviation. Multiple linear regression models for inflammatory markers adjusted for age, fasting status, smoking, hypertension, hypercholesterolemia, body mass index, census region, population density, case status, air pollution ($PM_{2.5}$), and use of anti-inflammatory medication. For NHS, models further adjusted for postmenopausal hormone use. Models for association between NDVI and inflammatory biomarkers were adjusted for nSES. Controls (n=10,061 for participants with at least one biomarker in NHS, n=4,481 for HPFS) results from models fit in sampled controls only. Non-movers results from models fit only in those participants who remained at the same address from 1986 (NHS, n=15,933) or 1988 (HPFS, n=7,423) through blood draw.

Supplementary Table 4. Associations between neighborhood contextual measures and inflammatory biomarkers in the Health Professionals Follow-up Study stratified by address type (home vs work)

	NDVI (270m)		nSES		Income ICE	
**Inflammatory marker**	**Percent change (95% CI)**	P_{het}	**Percent change (95% CI)**	P_{het}	**Percent change (95% CI)**	P_{het}
Adiponectin		0.27		0.13		0.93
Home	-0.77% (-4.36%, 2.96%)		0.05% (-2.78%, 2.97%)		2.49% (-1.61%, 6.76%)	
Work	2.11% (-1.85%, 6.24%)		3.38% (0.10%, 6.75%)		2.23% (-1.64%, 6.26%)	
CRP		0.51		0.45		0.14
Home	-1.60% (-7.28%, 4.43%)		-2.84% (-7.17%, 1.69%)		-2.81% (-8.96%, 3.77%)	
Work	1.16% (-5.11%, 7.85%)		-0.31% (-5.30%, 4.95%)		4.04% (-2.30%, 10.79%)	
IL-6		0.70		0.99		1.00
Home	-0.60% (-5.32%, 4.35%)		-1.81% (-5.40%, 1.90%)		-0.34% (-5.59%, 5.21%)	
Work	0.66% (-4.20%, 5.77%)		-1.77% (-5.64%, 2.24%)		-0.33% (-5.13%, 4.72%)	
sTNFR-2		0.76		0.48		0.57
Home	0.58% (-1.44%, 2.65%)		-2.70% (-4.21%, -1.16%)		-2.00% (-4.20%, 0.25%)	
Work	0.15% (-1.95%, 2.31%)		-1.91% (-3.58%, -0.21%)		-1.13% (-3.19%, 0.96%)	
Inflammation Score		0.89		0.42		0.18
Home	0.19% (-20.71%, 26.60%)		-25.95% (-38.00%, -11.56%)		-25.85% (-43.03%, -3.50%)	
Work	-2.00% (-22.90%, 24.57%)		-17.65% (-32.28%, 0.14%)		-5.53% (-25.72%, 20.13%)	

Abbreviations: CRP: C-Reactive Protein; IL-6: Interleukin-6; TNFR-2: soluble tumor necrosis factor receptor-2; nSES: Neighborhood Socioeconomic Status; ICE-Inc: Index of Concentration at Extremes-Income; NDVI: Normalized Difference Vegetation Index (NHS: 1986-1990, HPFS: 1990-1994), NHS: Nurses' Health Study; HPFS: Health Professionals Follow-up Study. Associations reflect an interquartile range increase in exposure. Multivariable linear regression models were adjusted for age, hypertension, hypercholesterolemia, nSES, BMI, census region, smoking, $PM_{2.5}$, and use of any anti-inflammatory medications. Covariates were assessed at blood draw.

Supplementary Table 5. Associations between NDVI (270m) within two years of blood draw and inflammatory markers by census region

Inflammatory marker	Percent change (95% CI)	P_{het}
NHS		
Adiponectin		0.12
Northeast	2.71% (0.58%, 4.86%)	
Midwest	-0.60% (-3.81%, 2.73%)	
South	-1.18% (-3.96%, 1.67%)	
West	1.93% (-1.28%, 5.23%)	
CRP		0.014
Northeast	-10.14% (-14.82%, -5.21%)	
Midwest	2.09% (-6.09%, 10.98%)	
South	-2.32% (-9.33%, 5.22%)	
West	1.44% (-6.49%, 10.04%)	
IL-6		0.0052
Northeast	-4.30% (-7.83%, -0.65%)	
Midwest	-0.03% (-5.69%, 5.97%)	
South	-9.27% (-13.84%, -4.46%)	
West	3.51% (-2.37%, 9.74%)	
sTNFR-2		0.78
Northeast	-0.80% (-2.30%, 0.72%)	
Midwest	0.60% (-1.82%, 3.08%)	
South	-0.21% (-2.28%, 1.92%)	
West	-0.92% (-3.28%, 1.52%)	
Inflammation score		0.082
Northeast	-31.65% (-43.76%, -16.95%)	
Midwest	5.33% (-22.51%, 43.16%)	
South	-15.03% (-35.31%, 11.61%)	
West	-9.86% (-33.38%, 21.96%)	
HPFS		
Adiponectin		0.39
Northeast	-0.61% (-6.01%, 5.12%)	
Midwest	0.64% (-4.34%, 5.90%)	
South	-0.12% (-4.22%, 4.16%)	
West	5.39% (-0.25%, 11.35%)	
CRP		0.73
Northeast	3.37% (-5.28%, 12.79%)	

Midwest	1.86% (-6.16%, 10.55%)	
South	-0.15% (-6.77%, 6.94%)	
West	-3.25% (-11.63%, 5.92%)	
IL-6		0.49
Northeast	3.73% (-3.53%, 11.53%)	
Midwest	2.58% (-3.85%, 9.45%)	
South	-2.40% (-7.73%, 3.23%)	
West	-1.17% (-7.89%, 6.04%)	
sTNFR-2		0.20
Northeast	1.36% (-1.72%, 4.52%)	
Midwest	0.59% (-2.16%, 3.41%)	
South	0.74% (-1.57%, 3.11%)	
West	-2.72% (-5.64%, 0.27%)	
Inflammation score		0.17
Northeast	17.48% (-17.11%, 66.53%)	
Midwest	20.15% (-11.97%, 64.00%)	
South	-9.61% (-31.12%, 18.64%)	
West	-22.81% (-45.31%, 8.94%)	

Abbreviations: CRP: C-Reactive Protein; IL-6: Interleukin-6; TNFR-2: soluble tumor necrosis factor receptor-2; nSES: Neighborhood Socioeconomic Status; ICE-Inc: Index of Concentration at Extremes-Income; NDVI: Normalized Difference Vegetation Index (NHS: 1986-1990, HPFS: 1990-1994), NHS: Nurses' Health Study; HPFS: Health Professionals Follow-up Study. Associations reflect an interquartile range increase in exposure. Multivariable linear regression models were adjusted for age, hypertension, hypercholesterolemia, nSES, BMI, census region, smoking, $PM_{2.5}$, and use of any anti-inflammatory medications. For NHS, models further adjusted for postmenopausal hormone use. Models for association between NDVI and inflammatory biomarkers were adjusted for nSES. Covariates were assessed at blood draw.
